# Tailoring Materials with Specific Wettability in Biomedical Engineering

**DOI:** 10.1002/advs.202100126

**Published:** 2021-08-08

**Authors:** Lingyu Sun, Jiahui Guo, Hanxu Chen, Dagan Zhang, Luoran Shang, Bing Zhang, Yuanjin Zhao

**Affiliations:** ^1^ Institute of Translational Medicine Department of Radiology The Affiliated Drum Tower Hospital of Nanjing University Medical School Nanjing 210002 China; ^2^ State Key Laboratory of Bioelectronics School of Biological Science and Medical Engineering Southeast University Nanjing 210096 China; ^3^ Zhongshan‐Xuhui Hospital the Shanghai Key Laboratory of Medical Epigenetics Institutes of Biomedical Sciences Fudan University Shanghai 200032 China

**Keywords:** biomaterials, biomedical engineering, slippery, superwetting, wettability

## Abstract

As a fundamental feature of solid surfaces, wettability is playing an increasingly important role in our daily life. Benefitting from the inspiration of biological paradigms and the development in manufacturing technology, numerous wettability materials with elaborately designed surface topology and chemical compositions have been fabricated. Based on these advances, wettability materials have found broad technological implications in various fields ranging from academy, industry, agriculture to biomedical engineering. Among them, the practical applications of wettability materials in biomedical‐related fields are receiving remarkable researches during the past decades because of the increasing attention to healthcare. In this review, the research progress of materials with specific wettability is discussed. After briefly introducing the underlying mechanisms, the fabrication strategies of artificial materials with specific wettability are described. The emphasis is put on the application progress of wettability biomaterials in biomedical engineering. The prospects for the future trend of wettability materials are also presented.

## Graphical abstract

As a fundamental feature of solid surfaces, wettability is playing an indispensable role in various fields such as academy, industry, agriculture, and biomedical engineering. This review comprehensively discusses the research progress of materials with specific wettability in biomedical engineering, ranging from underlying mechanisms, fabrication strategies, and application advances to their future prospects.

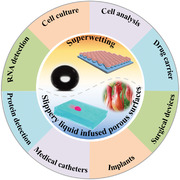

## 1 Introduction

As a common phenomenon in our daily life, the wettability of solid surfaces, which is determined by the interfacial interaction among solid, liquid, and gas phases, has attracted great attention and been extensively investigated. The concept of wettability and contact angle (CA) can be traced back to Young's equation proposed in 1805. Since then, a series of principles and models have been proposed successively, including Wenzel model and Cassie–Baxter model.^[^ ^1^, ^2^, ^3^ ^]^ According to these theories, intrinsic wettability states of the solid surfaces in air could be divided into four types including hydrophobicity, hydrophilicity, oleophilicity, and oleophobicity, where wettability could be influenced by the topographical structure and chemical composition of surfaces. With advances in theoretical researches and manufacturing technologies, various artificial surfaces with specific wettability have been developed to expand their applications, including superwetting surfaces and slippery liquid infused porous surfaces (SLIPS).^[^ ^4^, ^5^, ^6^, ^7^, ^8^, ^9^, ^10^ ^]^ Specifically, the possible states of superwetting surfaces involve superhydrophilicity, superhydrophobicity, superoleophilicity, superoleophobicity in air; superoleophilicity and superoleophobicity under water; superhydrophilicity and superhydrophobicity under oil. Because of their extraordinary wettability performance, the superwetting materials have greatly demonstrated practical importance. In addition, the *Nepenthes*‐inspired SLIPS also display excellent liquid‐repelling capacity, superiority in pressure stability, and optical transparency, which shows promising application prospects. As a popular topic in material science, researches on wettability not only facilitate the understanding of surface phenomenon and improve the materials’ properties, but also lend strong impetus to the development of other areas.

Up to date, wettability materials have been extensively applied in various fields involving academy, industry, agriculture, and biomedical engineering. Benefitting from the innovation and progress of preparation craft, wettability materials with different surface energy and morphologies, and even the intelligent ones with switchable wettability could be controllably fabricated by numerous methods such as 3D printing, template method, phase separation, spin‐coating, electrospinning, sol–gel method, and self‐assembly.^[^ ^11^, ^12^, ^13^, ^14^, ^15^, ^16^ ^]^ Thanks to these advances, the wettability materials have been employed in different applications depending on their own distinctive wetting performances. For example, the hydrophobic and oleophilic materials are usually applied for self‐cleaning, oil/water separation, anticorrosion, and anti‐icing fields;^[^ ^17^, ^18^ ^]^ the liquid‐infused surfaces could not only serve as anti‐icing and self‐cleaning materials, but also work for constructing optical devices;^[^ ^3^ ^]^ the oleophobic and hydrophilic surfaces show practical values in antifogging, antifouling, filtration, and biomedical fields;^[^ ^19^, ^20^, ^21^ ^]^ while hydrophobic and hydrophilic composite materials have the capacity of collecting water without energy input.^[^ ^22^ ^]^ Especially, the well‐designed superhydrophobic and superhydrophilic surfaces show unparalleled advantages in precise and lossless liquid manipulation,^[^ ^23^, ^24^, ^25^ ^]^ which further promotes the laboratory investigation and market‐oriented process of wettability materials.

With the increasing attention to healthcare, the practical values of wettability materials in biomedical‐related fields are receiving remarkable interests and researches.^[^ ^26^, ^27^, ^28^, ^29^, ^30^, ^31^, ^32^, ^33^, ^34^, ^35^, ^36^ ^]^ Previous literatures have revealed that surface wettability, especially the superwettability like superhydrophobicity and superhydrophilicity, have great influence on the biomolecular behaviors such as adhesion and proliferation.^[^ ^32^, ^33^, ^34^ ^]^ To be specific, the superhydrophobic surfaces are capable of effectively resisting bacterial adhesion, protein adsorption, cell adhesion, and blood coagulation; while the superhydrophilic ones have the ability of antibacterial and facilitating cell attachment. These features make the superwetting biomaterials appropriate for biomedical engineering applications including cell culture, biosensing and serving as implant materials.^[^ ^35^, ^36^ ^]^ In particular, the emergence of superwettability‐patterned surfaces provides an effective platform to regulate the physiological process.^[^ ^37^, ^38^ ^]^ Among them, the superhydrophobic–superhydrophilic patterned surfaces have demonstrated indispensable values on the micropatterning of the living cells for tissue engineering,^[^ ^39^ ^]^ cell‐based microarrays,^[^ ^40^ ^]^ and cellular fundamental research. In general, the superwetting biomaterials display great prospects in biomedical engineering field.

In addition to superwetting biomaterials, the SLIPS are also ideal candidates for biomedical applications. Generally, the SLIPS are fabricated by infiltrating lubricating fluids into various synthetic surfaces with micro‐ or nano‐textural roughness.^[^ ^6^ ^]^ Different from the traditional hydrophobic surfaces depending on solid–gas–liquid interfaces, the SLIPS realize the transition into solid–lubricant–liquid interfaces, which effectively improves the surface stability under external pressure. Based on the elaborate design, SLIPS demonstrate detect‐free and robust repellency to immiscible liquids of virtually any surface tension including the physiological fluids, proteins, microorganisms, and cells, making them suitable to serve as biomedical materials.^[^ ^41^, ^42^, ^43^ ^]^ To further increase the functionality, SLIPS with tunable wettability have been developed by introducing stimuli‐responsive elements to surfaces for dynamic manipulation of liquids, including response to thermal,^[^ ^44^ ^]^ electrical,^[^ ^45^ ^]^ magnetic,^[^ ^46^ ^]^ light,^[^ ^47^ ^]^ and pressure stimulations.^[^ ^48^, ^49^ ^]^ With these achievements, functional SLIPS have been applied in biomedical engineering such as cell culture and blood detection to construct medical instruments like medical tubing and implants, which provides new sight into addressing healthcare challenges.

Although great progress has been achieved in the research of wettability materials, especially for the biomaterials with specific wettability, their applications in biomedical engineering have seldom been reviewed. Herein, we will provide a comprehensive overview concerning wettability materials. After briefly introducing the fundamental theories of wettability, we will focus on the biomaterials with specific wettability, including the fabrications and their wettability performances. Examples of typical wettability biomaterials will be presented. Emphasis will be given to the application progress of wettability biomaterials in biomedical engineering, ranging from tissue engineering, biosensing to medical apparatus, and instruments. Finally, the perspectives and conclusions will be proposed to discuss the remaining challenges and the future development of wettability materials.

## 2 Fundamental Theories of Wettability

Wettability is a significant property of materials, which has great importance in various fields such as physics, chemistry, and biomedical engineering. In general, the wettability behavior at solid and liquid interfaces is determined by the surface morphology and its chemical composition. To evaluate the wettability performance, static CA, and contact angle hysteresis (CAH) are introduced as measurement indexes to reflect the static and dynamic wettability features of the materials, respectively. During the research process of wettability, a variety of classic theories and modern theories are put forward to clarify the mechanisms.^[^ ^1^, ^2^, ^50^, ^51^, ^52^, ^53^, ^54^, ^55^, ^56^, ^57^ ^]^ With the emergence of SLIPS in 2011, more advanced theories have been proposed to explain the wettability principle of this four‐phase system.^[^ ^3^, ^6^, ^56^ ^]^ Based on these theories, together with the development of material science, researchers have developed numerous materials with unique wetting phenomena to expand their practical applications. In this section, we will enumerate and explain the typical theories of wetting phenomena to make the concept clear, including the basic equations, measurement methods, and simple classification of materials with specific wettability.

### 2.1 Theoretical Models

#### 2.1.1 Young's Equation

**Figure 1:**
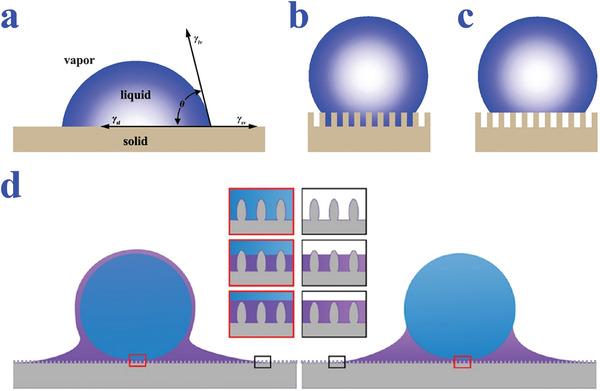
a) A liquid on an ideal solid surface (Young's mode). b) A liquid in contact with a rough surface (Wenzel's model). c) A liquid in non‐wetted contact with a rough substrate (Cassie's mode). a‐c) Reproduced with permission.^[^ ^47^ ^]^ Copyright 2010, The Royal Society of Chemistry. d) Different wetting states of the liquid‐infused surfaces. Reproduced with permission.^[^ ^56^ ^]^ Copyright 2018, Martin Villegas.

Nowadays, Young's equation is still the fundamental equation of wettability science since it was proposed by Thomas Young in 1805. The equation is established on the assumption that there is an ideal solid surface with smooth, inert, indeformable, and homogeneous properties, and the liquid droplet is identified as a consistent entity (**Figure** **1**a).^[^ ^52^ ^]^ When the liquid droplet is placed on the described surface, the system has an inevitable tendency to reach a balance status with lowest energy. Here, the surface tension referring to the specific energy at interfaces, is marked as *γ*, which reflects the cohesive strength of the underlying condensed phase. On the basis of the assumption, the wettability of materials could be characterized by Young's equation, that is

(1) $$\cos\theta_{\gamma }=\frac{\gamma_{\mathrm{sv}}\;-\;\gamma_{\mathrm{sl}}}{\gamma_{\mathrm{lv}}}\;$$

where *θ*_*γ*_ represents the equilibrium CA of the material. *γ*_sv_, *γ*_sl_, and *γ*_lv_ are interface tensions between solid and vapor (gas or air), solid and liquid, and liquid and vapor, respectively. According to the equation, it is not difficult to find out that the equilibrium CA depends on the interactions among three interfaces. However, Young's equation is a hypothetical and simplified mathematics model. In fact, the wettability of solid surfaces is also affected by other parameters such as roughness, heterogeneity, and areal deformation. Among them, surface roughness is a vital element for determining the CA value of surfaces. Taking this into account, Wenzel model and the latter Cassie–Baxter model are put forward to establish a relationship between roughness and the apparent CA.

#### 2.1.2 Wenzel Model

In Wenzel model which is presented in 1936, the liquids are considered as completely infiltrating the cavities and thus in contact with the entire rough surface (Figure 1b).^[^ ^52^ ^]^ Based on Young's equation, Wenzel introduced the concept of surface roughness, *r*, which referred to the proportion of the actual area to the apparent area of the surface. The modified equation is as follows:

(2) $$\cos\theta_{w}\;=r\cos\theta_{\gamma }\;$$

where *θ*_*w*_ and *θ*_*γ*_ represent the apparent CA and the intrinsic CA of the same material, respectively. Evidently, the value of *r* is greater than 1 for rough surfaces. From Equation (2), it could be derived that the surface roughness results in the amplification of wettability performance, that is, with the increase of surface roughness a hydrophilic surface will be more hydrophilic while a hydrophobic surface will become more hydrophobic. Compared with Young's equation, Wenzel model can explain a variety of wetting phenomena in nature and provide significant theoretical basis for the construction of wettability materials. However, according to the calculation of Equation (2), the cos *θ*_*w*_ of surfaces with extreme roughness would exceed 1, which is against mathematic principles. In addition, except for surface roughness, other parameters such as chemical heterogeneity also influence the CA of materials. Under the circumstances, Wenzel model is not applicable any more, and thus Cassie–Baxter model is further proposed to expand its feasibility.

#### 2.1.3 Cassie Model

Compared with Wenzel model, Cassie–Baxter model, which is also called Cassie model takes the chemical heterogeneities into consideration. To be specific, the liquid droplet in this model is incapable of filling all the voids distributed on the rough surface, because of the air pockets trapped underneath the liquid (Figure 1c).^[^ ^52^ ^]^ In this case, the contact system is composite and the air part is considered to be non‐wetting state. The resultant equation derived by Cassie and Baxter in 1944 can be described as:

(3) $$\cos\theta_{\mathrm{CB}}\;=\;f_{s}\cos\theta_{s}+f_{v}\cos\theta_{v}$$

where cos *θ*_CB_ is the apparent CA, while *f*_s_ represents the area fraction of solid and *f*_v_ signifies the area fraction of vapor on the rough surface, respectively. In this model, because *f*_s_  +  *f*_vs_ =  1, *θ*_s_  =  *θ*_*γ*_, and CA angle in air is almost equal to 180°, thus the Equation (3) can be simplified into:

(4) $$\cos\theta_{\mathrm{CB}}\;=\;-1+f_{s}\left(1+\cos\theta_{\gamma }\right)$$

By combining Equations (2) and (4), a more general equation to calculate the apparent CA of rough surfaces can be obtained, that is

(5) $$\cos\theta_{\mathrm{CB}}\;=\;-1+f_{s}\left(1+r\cos\theta_{\gamma }\right)$$

where *r* refers to the roughness degree of solid surface which is in contact with the liquid. It is worth mentioning that when *f*_s_ =  1, the Cassie equation can be converted into Wenzel model. Both Wenzel and Cassie model are valid for illustrating the wetting phenomenon on solid surfaces, on condition that the liquid droplet is large enough compared with the scale of roughness structure. The difference of Wenzel and Cassie model lies in the consideration on chemical heterogeneity. Under special conditions such as droplet press or vibration, the wettability behavior may switch from Cassie model to Wenzel state, which can be observed in practical terms.

#### 2.1.4 Other Models

Wenzel model and Cassie model have been recognized as theoretical basis for describing the wettability property of surfaces. However, these models are not applicable for all surfaces because the wetting state is complex and could be affected by various factors. Considering that, a variety of modified Wenzel and Cassie–Baxter models, such as Pease's model, Good's model, Patankar's model, and McHale model, have been developed in the past decades.^[^ ^50^, ^52^, ^53^, ^54^, ^55^, ^56^, ^57^, ^58^, ^59^, ^60^ ^]^ For example, Pease considered wettability as a 1D issue and created a line fraction‐based equation.^[^ ^53^ ^]^ Marmur's model revealed the possible transition between Wenzel and Cassie models.^[^ ^55^ ^]^ With the development of characterization equipment, Patankar et al. proposed a modified equation for hierarchical surfaces composed of micro‐scaled and nano‐scaled pillars.^[^ ^53^ ^]^ Afterward, Kwon et al. claimed that pillar‐shaped morphology did not comply with Cassie–Baxter model.^[^ ^53^ ^]^ Although various new theories have emerged, they are not widely accepted and still have limitations. Therefore, a more comprehensive and valid equation is still anticipated for studying the underlying mechanisms of wettability behavior.

With regard to slippery surfaces, Wenzel and Cassie–Baxter equations are incapable of explaining their liquid‐repellence. In this system, the wetting status is determined by the interaction among four phases including solid substrate, lubricating liquid, testing liquid, and gas.^[^ ^3^ ^]^ In order to simplify the model, the lubricating and testing liquids are assumed as fluorinated oil and water, respectively. The water droplet would be repelled by the lubricant in an encapsulated or meniscus‐shaped manner (Figure 1d),^[^ ^56^ ^]^ depending on the value of spreading parameter *S*_ow(a)_ that is defined as:

(5) $$S_{\mathrm{ow}\left(a\right)}=\gamma_{\mathrm{wa}}\;-\gamma_{\mathrm{ow}}-\gamma_{\mathrm{oa}}$$

where o, w, and a refer to oil, water, and air, respectively, and *γ* means the surface tension of two phases. When *S*_ow(a)_ > 0, the water droplet would be encapsulated by the lubricant oil; on the contrary, the droplet would lay on top. In addition to the surface tensions of liquids, the roughness of substrate also has impact on the wettability by affecting the penetration status of lubricant. Therefore, the selection of appropriate surface roughness and liquid system is important for tuning the wettability performance of slippery system.

### 2.2 Characterization of Wettability

Because wettability is an important property of materials, researchers have successively proposed a variety of characterization methods during the past centuries for evaluation and quantification. The most common measurements involving the static CA and the dynamic CA are utilized to fully reflect the surface property. Based on the liquid repelling behavior, together with the environment media, the wettability can be classified into various categories. Among them, materials with specific wettability achieve great attention for their outstanding performances and great potential for practical applications.

#### 2.2.1 Static Contact Angle

Since the concept of static CA is proposed, it has become an invaluable index to evaluate the wetting phenomenon, especially for the characterization of superwettability. In general, water droplets of 2–3 µL are suitable for obtaining the CA value of the solid surface, in which case the influence of gravity could be ignored. After recording the droplet morphology on the solid surface, followed by a fitting mode such as ellipse fitting, circle fitting, and Laplace–Young fitting, the value of CA can be easily achieved. During the measuring process, the CA value may differ from each other by utilizing different fitting models and using droplets of different volumes. Therefore, keeping the volume of test droplet constant is critical to ensure the accuracy of measurements. It is recognized that when CA is greater than 90°, the surface is regarded to be hydrophobic; when CA is less than 90°, the surface is considered as hydrophilic one. Especially, when CA is higher than 150°, the solid surface belongs to superwetting category called superhydrophobic status; when CA is less than 50°, the surface would be identified as a superhydrophilic one. Apart from water droplets, other liquids such as organic phases are also employed for evaluating the surface wettability. Similarly, according to the oil liquid morphology on solid substrate, there are four basic states including oleophilic, oleophobic, superoleophilic, and superoleophobic.

#### 2.2.2 Contact Angle Hysteresis

CAH, originating from the chemical and morphology inhomogeneity of the surface, can be used for describing the dynamic CA of the surfaces. Although static CAs have been extensively studied, they are incapable of reflecting the comprehensive wettability of materials. Several superhydrophobic surfaces that show a fairly high static CA could also make droplets difficult to slide down, like rose petals or rose petal‐inspired surfaces making droplets almost spherical without rolling off their surfaces.^[^ ^24^, ^53^, ^54^, ^61^ ^]^ With this regard, plenty of approaches have been invented to quantitatively measure the CAH value for reflecting wettability property from another perspective.^[^ ^2^, ^50^ ^]^ In fact, CAH refers to difference value between the advanced CA (*θ*_Adv_) and the receding CA (*θ*_Rec_). *θ*_Adv_ and *θ*_Rec_ are usually measured by an analysis software after the real‐time images recoded by a microscopy system. Specifically, when the droplet volume is gradually increased to move the contact line of three phases, the maximum CA at critical point is considered as *θ*_Adv_; when the droplet is slowly removed to change the three‐phase contact line, the minimum CA is defined as *θ*_Rec_. Essentially, the CAH phenomenon is generated from the kinetic barriers which prevent the droplets from achieving the minimum energy status depicted in Wenzel and Cassie model.

The CAH can also be reflected by tilt angle or sliding angle, that is, the critical angle of the droplet from fixing to moving upon inclining the solid surface. During the process, the rolling or sliding of droplets should be attributed to the gravity, and the corresponding sliding angle value usually ranges from 0° to 90°. When sliding angle is less than 10°, the water or other droplets could easily be removed from the surface, which indicates the self‐cleaning property of the material. If the solid substrate is smooth enough, as illustrated in Young's equation, the droplets would slide down under an extremely slight tilt. It is worth mentioning that the tilt angle value is not equivalent to the difference between *θ*_Adv_ and *θ*_Rec_. During the measurement process, tilt angle is affected by parameters incorporating the static sliding angle, CAH, and the volume of droplets. In most cases, both static CA and CAH are measured to characterize the wettability properties of materials.

#### 2.2.3 Classification of Wettability

Classified by the media, wettability can be roughly divided into three categories including the wettability in air, under water, and under oil.^[^ ^2^ ^]^ Among them, the wettability performance in air have become the emphasis of research for centuries. The four intrinsic states of the solid surfaces in air include hydrophobic, hydrophilic, oleophilic, and oleophobic. By taking inspiration from nature and introducing micro‐ or nano‐scale roughness, scientists have created plentiful artificial materials with specific wettability for various applications including superwetting materials, SLIPS, and wettability gradient surfaces. The superwetting behaviors of the surfaces contain superhydrophobic, superhydrophilic, superoleophilic, and superoleophobic states. When the environment turns into water or oil, the possible superwettability involve underwater superoleophobic, underwater superoleophilic, underwater superaerophobic, underwater superaerophilic, underoil superhydrophobic, underoil superhydrophilic, etc. These states in different media can be combined and the total amount of unique wettability states could reach up to 64 kinds.^[^ ^58^ ^]^ In particular, by integrating stimuli‐responsive materials or structure designs, these wettability states can be switched under control, thus further expanding their application values. Generally, the emergence and development of these wettability materials open a chapter in materials science and show great potential in practical applications.

## 3 Fabrication of Biomaterials with Specific Wettability

Nature is an endless treasury to mankind that provides considerable material paradigms with extraordinary wettability such as lotus leaf, water strider leg, spider silk, and butterfly wings.^[^ ^2^, ^6^, ^50^, ^58^ ^]^ Based on the inspiration from nature, researchers have developed numerous artificial surfaces with the desired properties for diverse fields.^[^ ^1^, ^2^, ^5^, ^6^, ^7^, ^10^, ^12^ ^]^ As chemical composition and roughness play crucial roles in determining the surface wettability, the modifications of the material wettability are around changing surface topography and chemical features.^[^ ^59^, ^60^ ^]^ With the development of science and technology, many advanced methods have been put forward to prepare materials especially biomaterials with specific wettability such as superwetting materials and SLIPS.^[^ ^52^, ^53^, ^54^, ^55^, ^56^, ^57^, ^58^, ^59^, ^60^, ^61^, ^62^, ^63^, ^64^, ^65^, ^66^, ^67^, ^68^, ^69^, ^70^, ^71^, ^72^, ^73^, ^74^, ^75^, ^76^, ^77^, ^78^, ^79^, ^80^, ^81^, ^82^, ^83^, ^84^, ^85^, ^86^, ^87^, ^88^, ^89^, ^90^, ^91^, ^92^, ^93^, ^94^, ^95^, ^96^, ^97^, ^98^, ^99^, ^100^, ^101^, ^102^, ^103^, ^104^, ^105^, ^106^, ^107^, ^108^, ^109^, ^110^, ^111^, ^112^ ^]^ In particular, numerous intelligent wettability surfaces have been developed by introducing stimuli‐responsive components which could in response to external stimuli including electric field,^[^ ^45^ ^]^ light,^[^ ^47^, ^113^, ^114^ ^]^ pH,^[^ ^115^ ^]^ magnetism,^[^ ^46^ ^]^ temperature,^[^ ^116^, ^117^ ^]^ stress, and ion,^[^ ^118^, ^119^, ^120^, ^121^, ^122^, ^123^, ^124^, ^125^ ^]^ greatly expand their application potential. Generally, the fabrication methods can be divided into physical methods, chemical methods, and the combination of physical and chemical methods.^[^ ^2^ ^]^ In the following section, we will put the emphasis on the fabrication process of biomaterials and their resultant wettability.

### 3.1 Physical Methods

Physical methods are widely used to fabricate superwettability materials, whose essence is altering the substrate roughness or depositing coatings of one new material on the physical plane. In other words, physical methods do not involve any chemical reactions. Up to date, the most common approaches contain 3D printing, template method, plasma treatment, spin‐coating ways and other methods.^[^ ^59^, ^60^, ^62^, ^63^, ^64^ ^]^ Based on the progress of manufacturing technology, countless biomaterials with extraordinary liquid‐repelling capacities have emerged, as shown in **Table** **1**.

**Table 1:** Representative fabrication methods of biomaterials with specific wettability

Fabrication methods	Material	Morphology	Wettability	Reference
Self‐assembly, photolithography, template method	PU, PEGDA, silicone oil	Hollow bump arrays	Slippery surface with hydrophilic patterns	^[^ ^7^ ^]^
Microfluidics, self‐assembly	ETPTA, SiO_2_, PNIPAM	Porous structure	Programmable wettability	^[^ ^10^ ^]^
Chemical polymerization	Graphene, TPI, perfluorinated oil	Porous structure	Tunable slippery property	^[^ ^45^ ^]^
3D printing	Photoresist IP‐S	Pillar structure	Superamphiphobic	^[^ ^68^ ^]^
3D printing	E‐glass, MWCNT	Eggbeater structure	Superhydrophobic, superolephilic	^[^ ^61^ ^]^
Template method	ETPTA, SiO_2_	Arch‐shaped microcavities	Anisotropic wettability	^[^ ^79^ ^]^
Nanoimprint lithography, plasma etching	FEP, PMMA, PET	Rough pillars	Superhydrophobic	^[^ ^82^ ^]^
Phase separation	PLA, SiO_2_	Nano/microstructured surface	Superhydrophobic	^[^ ^86^ ^]^
Spin‐coating, solvent annealing, plasma etching	PS‐b‐PDMS	Multilayer hierarchical structure	Superhydrophobic	^[^ ^89^ ^]^
Spray‐coating	HNTs, SiO_2_	Rod‐dot hierarchical structure coating	Superamphiphobic	^[^ ^92^ ^]^
Immersion treatment	Cu	Meshes with nanosheet structure	Superhydrophobic, superoleophilic	^[^ ^93^ ^]^
Electrospinning, chemical cross‐linking	PVDF/PEI‐EDA	Porous beads‐on‐string structure	Underwater oleophobic	^[^ ^95^ ^]^
Sol–gel methods	Silica	Hemispherical bumps	Hydrophobic layer with hydrophilic bumps	^[^ ^99^ ^]^
LBL method	TiO_2_, CuO	Nanoneedle‐like morphology	Superhydrophilic	^[^ ^102^ ^]^
Self‐assembly, CVD	SiO_2_, PS	Hierarchical photonic crystal structure	Superhydrophobic	^[^ ^104^ ^]^
fs laser, plasma polymerization	Borosilicate glass wafers	Double‐hierarchical surface structure	Superhydrophilic–superhydrophobic micropatterns	^[^ ^108^ ^]^
Anodizing, heating treatment	TiO_2_	Porous structure	Switchable superwettability in oil	^[^ ^119^ ^]^
fs laser, spin‐coating, thermal evaporation, condensation	Paraffin, ZnO, silver nanowire	Micropillar‐arrayed structure	Tunable slippery property	^[^ ^121^ ^]^
Hydrothermal synthesis, photosensitization, hydrophobization	ZnO, silicone oil	Nanorod arrays	Tunable slippery property	^[^ ^122^ ^]^

Polyurethane: PU; Poly(ethylene glycol) diacrylate: PEGDA; trimethylolpropane ethoxylate triacrylate: ETPTA; poly (*N*‐isopropylacrylamide): PNIPAM; Trans‐1,4‐polyisoprene: TPI; fluorinated ethylene propylene: multiwalled carbon nanotube: MWCNT; FEP; polymethyl methacrylate: PMMA; polyethylene terephthalate: PET; polylactic acid: PLA; polystyrene‐block‐polydimethylsiloxane: PS‐b‐PDMS; halloysite nanotubes: HNTs; polyetherimide: PEI; polyvinylidene fluoride: PVDF; ethanediamine: EDA; layer‐by‐layer: LBL; chemical vapor deposition: CVD; femtosecond: fs.

Among various physical methods, 3D printing is an emerging and revolutionary technology with the advantages of efficiency, controllability, and no need of complex procedure, which has attracted great interest and research in academia, industry and other areas since its appearance.^[^ ^61^, ^65^, ^66^, ^67^, ^68^, ^69^ ^]^ With the progress of manufacturing, 3D printing has been applicable for fabricating wettability surfaces with nanoscale features by laser polymerization,^[^ ^65^, ^66^, ^67^ ^]^ which is also called direct laser writing or two‐photon laser writing. Based on 3D printing, wettability surfaces with different patterns such as pillar structure,^[^ ^68^ ^]^ eggbeater‐like structure,^[^ ^61^ ^]^ and spine structure^[^ ^69^ ^]^ have been developed and demonstrated superwetting performance. By adjusting printing parameters, the liquid‐repelling capacity of the derived surfaces would change correspondingly. Generally speaking, 3D printing is an ideal candidate for manufacturing wettability biomaterials with high‐resolution pattern designs. However, the defects of being expensive and time‐consuming limit its further applications.

Template‐based methods have become versatile and feasible choices to prepare the surfaces with specific wettability, since the concept of template method was proposed in 1990s.^[^ ^54^ ^]^ In this method, surface roughness, which relates to the wettability performance of materials, can be adjusted by employing different templates with different pattern designs. Various micro‐ or nano‐patterns have been developed such as conical, pillar‐structured, honeycomb‐like, and textured.^[^ ^70^, ^71^, ^72^, ^73^, ^74^, ^75^, ^76^ ^]^ In general, the template method involves three procedures: the preparation of templates with featured morphology, the molding process, and the removal of templates. As a consequence, the replication samples would finally present the same structure with the templates. Templates could be biological materials in nature such as flora and fauna surfaces.^[^ ^77^, ^78^ ^]^ Except for biological samples, templates can also be artificial surfaces with the aid of progressive techniques such as lithography, ferrofluids, and microfluidic approaches.^[^ ^79^, ^80^ ^]^ Because of the advantages of simplification, cheapness, effectiveness, and mass production, the template method has been extensively utilized and developed. However, the repeatability of template method is not ensured because the removal of the templates may damage the samples and even the templates themselves. In addition, the peeling off process makes the template methods only applicable for replicating relatively simple and regular patterns.

In addition to template method, plasma treatment is another common but effective way to obtain superwetting surfaces.^[^ ^81^ ^]^ Generally, plasma treatment contains plasma etching and plasma polymerization to obtain materials with roughness and coatings. Plasma etching is able to create micro‐ or nano‐structures to increase the roughness of substrates.^[^ ^82^, ^83^ ^]^ Different from plasma etching, plasma polymerization depends on monomers with superwetting behaviors in the gas phase to decorate surfaces.^[^ ^84^, ^85^ ^]^ The plasma treatment has the advantages of being rapid and highly selective, but its further popularization is limited by the expensive instruments and low output. Phase separation method is one of the conventional physical methods to fabricate wettability materials based on the instability of multicomponent mixture. In brief, the instable mixture is separated into phases under certain conditions like sudden cooling or heating, thus forming a bicontinuous structure to increase the surface roughness. Thanks to the superiority in low cost and feasibility, phase separation has been extensively utilized in fabricating superwetting materials, especially superhydrophobic surfaces.^[^ ^86^, ^87^ ^]^

Spin‐coating and spray‐coating are also traditional choices to manufacture biomaterials with specific wettability, through forming a thin polymer or nanoparticle film on the surfaces. In spin‐coating method, the solution or suspension is coated on the substrates and then spun into uniform thickness.^[^ ^88^ ^]^ Spin‐coating method shows advantages in easy preparation and low cost but is merely suitable for flat substrates, which limits its further application. To enhance the superwetting behavior of materials, spin‐coating technique is usually combined with other physical methods.^[^ ^89^, ^90^ ^]^ Similar to the spin‐coating method, spraying method is also aimed at decorating the surfaces with a low surface energy layer to impart surfaces with enhanced wettability, through spraying and subsequently solidifying the solutions on substrates.^[^ ^91^ ^]^ During the spray‐coating process, a glue layer is usually sprayed on the substrate before the spray of the nanoparticle suspensions to enhance the adhesion of functional particles.^[^ ^92^ ^]^ The spraying method shows advantages in simple operation, versatility, and no restrictions on the substrates, making it suitable for large‐scale industrial production. Apart from the methods mentioned above, various other physical methods have also been presented to endow biomaterials with specific wettability, such as dip‐coating, electrospinning, and physical vapor deposition.^[^ ^2^, ^93^, ^94^, ^95^, ^96^ ^]^ With the development of material science, it could be predicted that more facile, effective, and costless physical methods would emerge in future.

### 3.2 Chemical Methods

Different from physical methods, chemical treatment process usually involves the chemical reactions such as etching, polymerization, and molecular grafting, which shows advantages of being simple, rapid, and effective. So far, the popular chemical methods for surface modification include sol–gel method, layer‐by‐layer technique, self‐assembly, and chemical vapor deposition (CVD).^[^ ^2^, ^97^, ^98^, ^99^, ^100^, ^101^, ^102^, ^103^, ^104^, ^105^, ^106^, ^107^, ^108^, ^109^, ^110^, ^111^, ^112^, ^113^, ^114^, ^115^, ^116^, ^117^, ^118^, ^119^, ^120^, ^121^, ^122^, ^123^, ^124^, ^125^ ^]^ With decades of development, these methods have achieved great progress in fabricating various wettability materials in precise and controllable manner, paving the way for further application of these materials.

Sol–gel method is a typical chemical strategy for surface modification, which has made success in fabricating superhydrophilic, superhydrophobic, and superoleophobic surfaces. In this method, the substrates are usually immersed in a precursor solution or sol containing active monomers, followed by dry or heat treatment to form oxides or other composite network on the surfaces. By changing the components of the precursor solution, the roughness of the resultant surfaces could be easily adjusted.^[^ ^97^, ^98^ ^]^ In addition to fabricating surfaces with uniform wettability performance, sol–gel method is also suitable for manufacturing materials with composite wettability.^[^ ^99^ ^]^ Layer‐by‐layer (LBL) technique has evolved into a relatively mature method for fabricating multilayered films with specific wettability, which depends on the electrostatic interaction among different layers such as polyanion and polycation.^[^ ^100^, ^101^, ^102^ ^]^ To be specific, the positively and negatively charged layers are alternately deposited on the substrates until reaching the desired thickness. The LBL technique is convenient and economical, and has no restrictions on the substrates, thus receiving great attention. Up to date, LBL assembly has been widely utilized in the preparation of superhydrophobic surfaces based on various substrates ranging from glass, plastic to fabrics, thus greatly expanding the application fields.^[^ ^2^, ^100^ ^]^ In addition, LBL assembly is also applicable for fabricating superhydrophilic surfaces with various architectures such as silica hollow spheres and strawberry‐like composites.^[^ ^101^, ^102^ ^]^

Self‐assembly method refers to the spontaneous assembly of molecular and nanoscale units to form the desired roughness on substrates. The self‐assembly is a common method to fabricate wettability materials, whose process depends on noncovalent interactions such as van der Waals force,^[^ ^103^, ^104^ ^]^ thus providing a simple way for the fabrication of surfaces with controllable wettability. CVD is also a promising method to fabricate superwetting materials due to its capacity of constructing specific nanostructures in a controllable manner.^[^ ^64^, ^105^, ^106^ ^]^ During the process, the substrates are usually deposited with the wettability layer by exposing to a gaseous precursor for chemical reaction.^[^ ^105^ ^]^ In previous studies, CVD has been extensively utilized to fabricate superhydrophobic materials with different nanostructures, such as honeycomb‐like, pillar structured, and island‐shaped carbon nanotube patterns.^[^ ^64^ ^]^ Numerous efforts have been devoted to improving the stability of the resultant surfaces for further applications via choosing materials with excellent mechanical and chemical properties.^[^ ^106^ ^]^

As a simple and cheap technique, etching has been widely used in possessing the surfaces to gain roughness. Based on the chemical nature of substrates, different etching methods could be applied such as ion‐etching, solution etching, and photolithography. Benefitting from the progress of these techniques, various wettability materials have been successfully fabricated.^[^ ^107^, ^108^, ^109^ ^]^ Except for the abovementioned methods, many other techniques have also been explored to manufacture wettability materials, such as hydrothermal treatment, calcination, and grafting. In fact, these methods are usually integrated together, and even combined with physical methods for better control over the surface textures as well as chemical compositions.^[^ ^110^, ^111^, ^112^ ^]^ In general, these methods achieve great progress in fabricating wettability materials while each method has their own advantages and disadvantages. Researchers may choose the most appropriate one or integrate these methods according to practical requirements to produce the desired surfaces.

## 4 Biomaterials and Tissue Engineering

Depending on different surface topography and chemistry design, wettability biomaterials have been developed and showed great influence on regulating biomolecular behaviors such as cell adhesion, protein adsorption, and microorganism resistance. Based on these features, wettability biomaterials have found wide application prospects in biomedical‐related fields.^[^ ^35^, ^41^, ^126^, ^127^ ^]^ In this section, we will review the cutting‐edge progress of biomaterials with specific wettability in tissue engineering, ranging from their effects on cell culture and analysis to roles as drug carriers and contrast agents.

### 4.1 Cell Culture and Analysis

The control over cell behaviors including cell adhesion, spreading, shaping, orientation, migration, and differentiation has great significance in tissue engineering and other life science applications. Previous literatures have shown that wettability materials can efficiently manipulate cell behaviors by tuning their surface topography and chemical components.^[^ ^32^, ^33^, ^34^ ^]^ Benefitting by the development of cell biology and material science, 2D cell culture on various wettability biomaterials have been successfully constructed in a controllable manner.^[^ ^42^, ^43^, ^128^, ^129^, ^130^, ^131^, ^132^, ^133^, ^134^, ^135^ ^]^ In addition to 2D cell cultivation, the wettability biomaterials also show superiority in culturing 3D organoids, significantly to expand their biomedical applications.^[^ ^136^, ^137^, ^138^, ^139^, ^140^, ^141^, ^142^, ^143^ ^]^ Based on these features, the biomaterials with cell adhesion have displayed potential for constructing high throughput cell‐based assays.^[^ ^144^, ^145^, ^146^, ^147^, ^148^ ^]^ By further integrating the wettability materials cultured with cells into microfluidic chips, the biohybrid system could serve as lab‐on‐a‐chip system for drug evaluation and other applications.^[^ ^149^, ^150^, ^151^, ^152^ ^]^

#### 4.1.1 Two‐Dimensional Cell Cultivation

As a fundamental and critical role in cell biology, 2D cell cultivation has emerged as a relatively mature system for cellular mechanisms studying, drug evaluation, and even tissue implants constructing. Since the wettability materials have demonstrated values in regulating cell behaviors, plenty of 2D cell culture systems have been established based on different biomaterials, especially those with specific wettability. In general, the superhydrophobic surfaces with a Cassie–Baxter state usually show favorable repellence to cell adhesion. For example, Ko et al. investigated the behaviors of mouse liver cancer cells on the superhydrophobic surfaces prepared by oxygen plasma ion etching.^[^ ^129^ ^]^ The results revealed that superhydrophobicity property effectively suppressed the adhesion and growth of cancer cells by maintaining their spherical shape, which offered a new idea to control the cell behaviors. On the contrast, the superhydrophilic materials are found to have effect on facilitating the attachment of cells, as well as promoting their proliferation, migration, differentiation, and other physiological processes.

**Figure 2:**
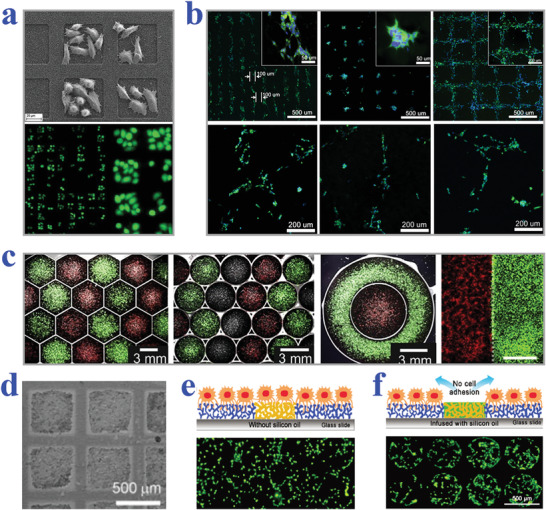
a) SEM (top) and fluorescence (bottom) images showing CHO cells trapped within square superhydrophilic regions. Reproduced with permission.^[^ ^130^ ^]^ Copyright 2011, The Royal Society of Chemistry. b) Fluorescent patterns of “K,” “I,” and “T” formed by neuroblastoma cells. Reproduced with permission.^[^ ^132^ ^]^ Copyright 2016, Elsevier. c) Merged bright‐field and fluorescence micrographs demonstrating different geometries patterned with different cells. Reproduced with permission.^[^ ^133^ ^]^ Copyright 2012, Elsevier. d) Bright field image of HEK 293 cells cultured on a slippery surface with square patterns (500 µm side length and 100 µm barrier). Reproduced with permission.^[^ ^42^ ^]^ Copyright 2013, Wiley ‐VCH. e,f) Schematic diagram and fluorescent images of cell adhesion situation on superhydrophobic barriers (e) and silicone‐oil‐infused superhydrophobic barriers (f). Reproduced with permission.^[^ ^43^ ^]^ Copyright 2016, The Royal Society of Chemistry.

Based on these features, superhydrophobic and superhydrophilic patterned wettability surfaces are capable of effectively controlling the micropatterning of cells. For example, Piret et al. realized selective mammalian cell adhesion to the superhydrophilic regions by culturing Chinese Hamster Ovary K1 (CHO) cells on patterned superhydrophilic/superhydrophobic silicon nanowire (SiNW) surfaces, as shown in **Figure** **2**a.^[^ ^130^ ^]^ During the process, the dissolution of superhydrophilic nanowires with cell adhesion and proliferation was observed, which may be attributed to the strong interaction between cells and the hydrophilic substrate; while the superhydrophobic area showed great resistance to cell adhesion and culture medium. Similarly, Marcon et al. constructed a kind of square‐structured cell micropatterning on superhydrophilic/superhydrophobic diamond nanowire surfaces in a controllable manner.^[^ ^131^ ^]^ Apart from square‐shaped structures, various wettability patterns with different designs have also been developed for cell culture. Ishizaki et al. successfully realized circular and rectangular cellular micropatterning based on superhydrophilic/superhydrophobic surfaces by using NIH 3T3 fibroblast cells.^[^ ^38^ ^]^ In another work, Park et al. developed line, circle, mesh, and even letter‐shaped cell patterns by selectively decorating nanocrystalline diamond surfaces to gain wettability difference, as shown in Figure 2b.^[^ ^132^ ^]^ The controllable manipulation of cellular behaviors based on wettability patterned biomaterials was anticipated to facilitate the development of cell‐based biosensors or even tissue repair and regeneration. In fact, the biological processes of cells in vivo are much more complicated, which usually involve complex geometric construction and effective communication among various cell types. With this regard, Efremov et al. designed a micropatterned substrate with highly hydrophilic regions separated by superhydrophobic borders for the coculture of multiple cell lines, as shown in Figure 2c.^[^ ^133^ ^]^ Based on the wettability‐patterned substrates, multiple cell types such as kidney HEK 293, HeLa, and NIH‐3T3 fibroblast cell lines were simultaneously cultured to form complex cellular patterns with different geometry designs. Although the cell migration among the hydrophilic regions was prevented by superhydrophobic borders, the cells still realized effective cell communication without direct contact depending on signaling molecules Wnt8.

Similar to superhydrophobic materials, slippery surfaces also exhibit great resistance to cell adhesion, owing to their stable liquid‐repelling capacity. By taking advantage of this feature, Ueda et al. proposed a hydrophobic liquid‐infused porous polymer surface based on superhydrophilic/superhydrophobic substrates for repelling cells in an efficient and stable manner.^[^ ^42^ ^]^ The as‐described surface was constructed by selectively infusing lubricant into superhydrophobic regions through blocking superhydrophilic regions with water, which showed favorable stability no matter in solution or air. When the hydrophobic liquid micropatterns were employed for cell culture, cells would be restricted in superhydrophilic regions by hydrophobic barriers to eventually form predesigned geometries, as shown in Figure 2d. Analogously, Shi et al. demonstrated a strategy for constructing cell micropatterns on silicone‐oil‐modified slippery surfaces.^[^ ^43^ ^]^ The superhydrophilic spots achieved from UV irradiation under photomask were separated by silicone‐oil‐infused superhydrophobic barriers. To verify the cell‐repelling capability of the resultant surfaces, their performance was compared with the air‐assisted superhydrophobic substrates without oil modification. It was demonstrated that the NIH‐3T3 cells cultured on the superhydrophilic/superhydrophobic surface did not form regular shapes; while circle‐shaped cellular micropatterning was clearly observed on the silicone‐oil‐modified wettability surfaces by choosing appropriate size of barriers (Figure 2e,f). To better mimic the multicellular environment in vivo, NIH‐3T3 and MCF‐7 cells were successfully cocultured on the superhydrophilic regions (400 µm wide) separated by silicone‐oil‐infused superhydrophobic barriers (100 µm) without migration.

In particular, biomaterials with tunable wettability make the controllable capture and release of cells possible, opening a new chapter for the investigation of cellular behaviors. It has demonstrated that the existence of hydrophobic interaction could facilitate cell adhesion on hydrophobic surfaces and suppress cell interaction with hydrophilic surfaces. Based on this principle, Cui et al. realized dynamic control of cell adhesion in response to near‐infrared (NIR) by grafting thermo‐responsive hydrogel poly(*N*‐isopropylacrylamide) on SiNWs.^[^ ^134^ ^]^ Because of the photothermal effect of SiNWs, the NIR irradiation could be efficiently transferred into heat to induce the phase transformation of hydrogel, in which case the hydrophilic state turned into hydrophobic performance, thus enhancing the cell adhesion. By adjusting the “on” or “off” status of NIR, the wettability could be correspondingly switched to realize the reversible adhesion and release of cells. Attractively, once the biomaterial was further decorated with antibody against epithelial‐cell adhesion molecule, the surface could specifically capture and release MCF‐7 cells under non‐invasive NIR control. In addition, Wei et al. developed an electrochemically wettability‐switchable surface that altered by nanotube‐ and nanotip‐shaped polypyrrole arrays to mediate the fate of mesenchymal stem cells.^[^ ^135^ ^]^ The cyclic attachment and detachment of stem cells, which were controlled by the switch between hydrophobic nanotubes and hydrophilic nanotips, were proved to have great influence on promoting the progress of intracellular mechanotransduction and osteogenic differentiation. Although significant strides have been made in 2D cell cultivation on wettability materials, it is still challenging to mimic in vivo physiological environments.

#### 4.1.2 Three‐Dimensional Cell Culture

**Figure 3:**
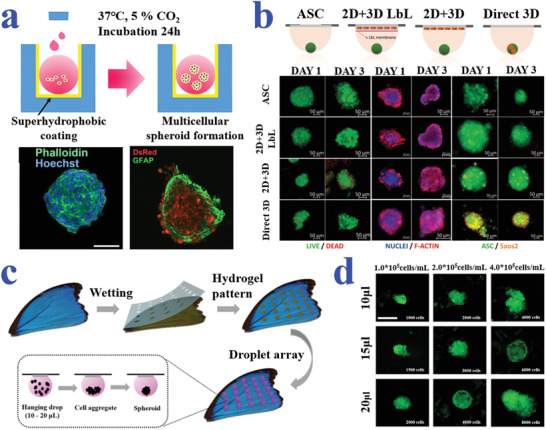
a) Schematic and fluorescence images of cell spheroid formation in NLM system created by superhydrophobic coating. Reproduced with permission.^[^ ^140^ ^]^ Copyright 2019, American Chemical Society. b) Schematic diagram and representative fluorescence microscopy images of the different configurations for spheroids formed by hanging drop strategy. Reproduced with permission.^[^ ^142^ ^]^ Copyright 2017, Wiley‐VCH. c,d) Schematic diagrams and fluorescent images illustrating the formation of cell spheroids on the superhydrophobic butterfly wing with hydrophilic hydrogel spots. The scale bar in (d) is 500 µm. Reproduced with permission.^[^ ^143^ ^]^ Copyright 2019, American Chemical Society.

Compared with traditional 2D cell cultivation, 3D cell culture provides a more effective strategy to study the complex cellular interactions by constructing in vivo like microenvironments. More importantly, the 3D‐cultured cell tissues may reproduce similar physiological structures or even present partial functions of specific organs in vitro, thus achieving extreme attention and research.^[^ ^136^, ^137^, ^138^, ^139^ ^]^ An astonishing variety of methods have been developed to generate cell spheroids, such as magnetic levitation, hanging drop techniques, and microfluidic platforms. Among them, wettability materials especially the superhydrophobic surfaces have been widely utilized for facilitating the formation of cell spheroids, owing to their extraordinary capacity of anti‐adhesion capacity. For example, Chen et al. constructed a 3D cell culture system based on naked liquid marbles (NLM) by decorating superhydrophobic coatings on microplates where the cultured medium droplets presented spherical shape, as shown in **Figure** **3**a.^[^ ^140^ ^]^ In this research, mouse olfactory ensheathing cells (mOECs) were initially chosen as the model cells to investigate the effect of NLM system on forming spheroids. The results showed that uniform mOECs spheroids could be generated with size showing a positive relationship with cell‐seeding density. In addition, human neural progenitor cell spheroids, as well as cocultured Schwann cells and astrocytes spheroids with favorable viability also formed in NLM system, demonstrating its feasibility in 3D cell cultivation.

In particular, the combination of the hanging drop system with patterned‐wettability materials has achieved great progress in generating cell spheroids, understanding relevant mechanisms and finding applications in tissue engineering and drug development. For instance, Oliveira et al. suggested superhydrophobic surfaces with wettable regions to serve as substrates for high‐throughput cultivation of cell spheroids by integrating the hanging drop technique.^[^ ^141^ ^]^ In the following work, they utilized patterned superhydrophobic platforms for coculturing multiple cell lines to produce stem‐cell spheroids, with the purpose of better imitating in vivo microenvironment.^[^ ^142^ ^]^ The coculture hanging drop system was conductive to generate human adipose‐derived stem cell microtissues in the either direct or indirect contact with other cells, as shown in Figure 3b. Shao et al. described the formation of cell spheroids on naturally superhydrophobic butterfly wing with hydrophilic hydrogel arrays (Figure 3c).^[^ ^143^ ^]^ Based on the composite substrate, different volumes of droplet microarrays could be hanged by adjusting the diameter of hydrogel patterns for cell cultivation (Figure 3d). Thanks to the excellent hydrophobicity of the wing substrate, droplets could maintain their spherical shape with minimized spreading degree. It was demonstrated that HepG2 (human hepatocarcinoma cell line) spheroids were successfully produced by the hanging drop method, which showed potential for investigating tissue‐level biology and drug screening.

#### 4.1.3 Cell‐Based Assay

**Figure 4:**
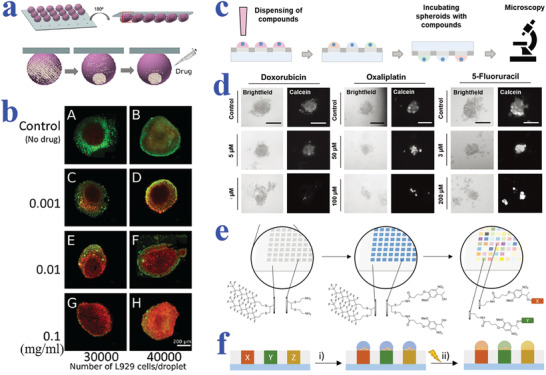
a) Schematic diagram showing the formation of cell spheroid and subsequent drug‐screening on patterned superhydrophobic surfaces. b) Fluorescent images of L929 spheroids added with different concentrations of DOX. a,b) Reproduced with permission.^[^ ^144^ ^]^ Copyright 2014, Wiley‐VCH. c) Screening of compounds based on single‐spheroid‐microarrays. d) Microscopy images of HeLa spheroids incubated with different types and different concentrations of drugs. Scale bars are 100 µm. c,d) Reproduced with permission.^[^ ^146^ ^]^ Copyright 2019, Wiley‐VCH. e) Schematic illustration of the high‐throughput solid‐phase synthesis on hydrophilic–superhydrophobic substrates. f) Scheme showing the release of synthesized compounds under UV irradiation (365 nm) for cell‐based screening. e,f) Reproduced with permission.^[^ ^148^ ^]^ Copyright 2020, Wiley‐VCH.

Benefitting from the superiority in high‐throughput generation of cell layers or cell spheroids, wettability materials show potential in replacing traditional microplates for cell‐based screening such as drug evaluation, gene expression analysis, and other chemical or biological molecules investigation. Among them, the application of cell‐based assay in drug evaluation has been widely explored to provide guide for clinical treatment, relying on the advance in wettability patterned substrates. In particular, the formation of tumor spheroids could facilitate the understanding of cancer‐related mechanisms and the development of antineoplastic drugs. Neto et al. employed the superhydrophobic substrate with micro‐indentations to form mouse lung fibroblast cell line L929 spheroids for drug screening, as shown in **Figure** **4**a,b.^[^ ^144^ ^]^ The cell aggregates were formed by gravity through hanging droplets containing cells on the inverted wettability platform. Different doses of anticancer drug doxorubicin (DOX) were then injected into the droplets to evaluate the corresponding therapeutic effect. The cell viability showed a negative relationship with the DOX concentration, which was in accordance with the expectation. In addition, higher cell death rate was observed in larger spheroids, which may be attributed to the insufficient nutrient supply and waste exchange.

Popova et al. developed a droplet‐array sandwiching technique to culture cells, and then conducted drug screening on the superhydrophilic/superhydrophobic patterned substrates.^[^ ^145^ ^]^ In the further work, the same team developed droplet microarrays based on superhydrophobic substrates with hydrophilic regions as platforms to cultivate tumor spheroids for high‐throughput drug screening (Figure 4c,d).^[^ ^146^ ^]^ On account of the separated droplet units, the miniaturized droplet array platform was suitable for carrying out individual biological and chemical experiments. To verify this feature, multiple anticancer compounds including DOX, oxaliplatin, and 5‐fluoracil were applied to treat HeLa cell spheroids and 2D HeLa layer. Both 2D and 3D cell formats displayed dose‐response to the anticancer compounds, that is, the cell viability decreased with the increase of drug concentration. In addition, 3D cell spheroids showed more resistance to drugs compared with 2D cell monolayer, which was identified with the previous reports. Except for screening conventional drugs, the cell‐based wettability platforms are also capable of synthesizing novel compounds and then evaluating their biological responses. Benz et al. demonstrated 75 parallel synthesis of lipidoids on a wettability‐patterned chip, followed by a high throughput biological screening.^[^ ^147^ ^]^ In the next research conducted by the same group, Brehm et al. also employed the miniaturized droplet microarrays formed on the superhydrophobic background with hydrophilic spots to perform a simultaneously combinatorial synthesis of over 500 compounds and cell‐based screening, as shown in Figure 4e,f.^[^ ^148^ ^]^ The solid‐phase synthesis was based on the four‐component Ugi reaction and a photocleavable linker as anchor point, thus imparting the compounds with photo‐controlled release capacity. After irradiation by UV light, the synthesized compounds could be released to the droplets with cell adhesion for biological screening.

**Figure 5:**
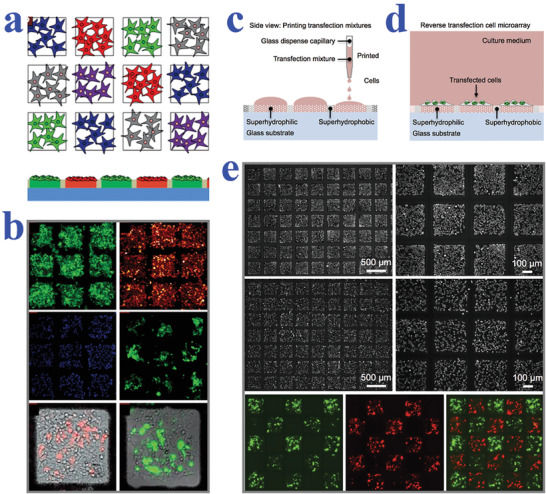
a) Schematic representation of isolated transfected cell clusters in microspots without migration and cross‐contamination. b) Fluorescent images of four different cell lines cultured on the array and HEK cells transfected with different plasmids on two superhydrophilic regions. a,b) Reproduced with permission.^[^ ^149^ ^]^ Copyright 2011, Wiley‐VCH. c,d) Schematic diagrams of the reverse transfection procedure using the superhydrophilic/superhydrophobic patterned surfaces. e) Microscopy images showing HEK 293 cells and HeLa cells confined in square superhydrophilic spots; fluorescent images of the transfected cells showing minimal cross‐contamination between neighboring regions. Reproduced with permission.^[^ ^150^ ^]^ Copyright 2016, Wiley‐VCH.

In recent decades, patterned wettability materials are attaching increasing interest in genetic analysis area for their capacity of constructing cell arrays in an isolated and parallelized manner. For example, Geyer et al. described a simple strategy to construct cell microarrays based on superhydrophilic spots surrounded by superhydrophobic barriers, as shown in **Figure** **5**a,b.^[^ ^149^ ^]^ Because of the wettability contrast, the cell migration and cross‐contamination could be successfully prevented by adjusting the superhydrophobic gaps. After treating the wettability‐patterned platform with gelatin contained transfection regents, the cells were seeded on the obtained array to uptake the nucleic acids for investigating genomic functions, which set a solid foundation for the development of genome‐on‐a‐chip. To rapidly screen the lipid libraries, as well as better understand the transfection mechanisms, Ueda et al. utilized cell microarrays based on superhydrophilic/superhydrophobic substrates for transfection with characteristics of ultrahigh throughput and less regent consuming (Figure 5c–e).^[^ ^150^ ^]^ To be specific, supposing that the side length of superhydrophilic squares is 335 µm and the width of superhydrophobic barrier is 60 µm, at least 6000 cell arrays could be simultaneously formed on the glass slide. ScreenFect A, a typical liposomal transfection reagent, was then chosen to verify the transfection efficiency and optimize the experimental parameters including initial cell density, incubation time, and DNA concentration. The results demonstrated the feasibility of the cell‐based assays for screening lipid and other chemical libraries. Besides, Bian et al. developed an electroporation system integrated with superhydrophobic microwell chip to construct a high throughput platform, which realized effective delivery of single guide RNAs into mammalian cells.^[^ ^151^ ^]^

Apart from drug evaluation and gene expression analysis, the cell arrays formed on patterned wettability surfaces also exhibit potential in screening biological molecules such as stem cells. For instance, Tronser et al. reported nanoporous superhydrophobic‐hydrophilic patterns for high‐throughput screening of stem cells based upon poly(2‐hydroxyethyl methacrylate‐*co*‐ethylene dimethacrylate) substrate, which had advantages of modifiable surface property and inhibiting mouse embryonic stem cell (mESC) differentiation without the existence of mouse embryonic fibroblasts.^[^ ^152^ ^]^ By applying a transgenic mESC line that expressed enhanced green fluorescent protein, the stemness could be directly read out via measuring the fluorescence or directly counting the cell colonies. According to the results, the differentiation of stem cells on the wettability‐patterned surface was inhibited up to 72 h, which may be caused by the nanoscale roughness. In this case, the proposed platform was suitable for screening mESCs for further applications in regenerative medicine or tissue engineering. In general, cell‐based assay provides a highly efficient platform to accelerate the progress of chemical and biological discovery, as well as significantly reduces the research and development costs.

### 4.2 Drug Carriers

Drug delivery, referring to the effective transportation of therapeutic drugs to specific parts or organs, has been extensively researched to promote the development of medicine.^[^ ^153^, ^154^, ^155^ ^]^ The main challenges of constructing an effective drug delivery system involve addressing poor water‐solubility of drugs, improving targeting capacity, protecting drugs from being attacked by the immune system, and realizing controllable and long‐term release. Various drug delivery systems based on different carriers such as lipids and micelles have been developed to solve the abovementioned problems. In particular, the introduction of wettability materials improves the performance and thus expands the practical applications of drugs carriers, benefitting from their specific interactions with drugs and release media. Additionally, smart drug carriers based on wettability‐tunable materials have also been developed to facilitate the development of novel drug delivery systems.

**Figure 6:**
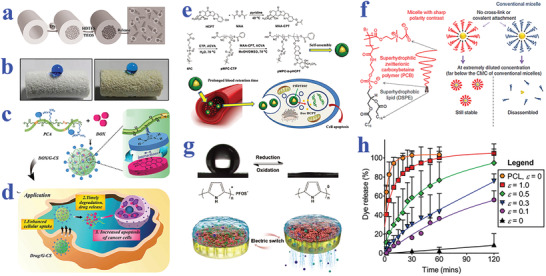
a) Schematic illustration of DS loading, superhydrophobic polyorganosilanes modification, and air cushion for controllable release. Reproduced with permission.^[^ ^159^ ^]^ Copyright 2014, Wiley‐VCH. b) Optical images of superhydrophobic polymeric matrix before and after loading tetracycline molecules. Reproduced with permission.^[^ ^160^ ^]^ Copyright 2018, The Royal Society of Chemistry. c) Schematic showing the construction of a drug delivery system based on the assembly of DOX and biodegradable hydrophilic poly(agmatine). d) Schematic showing the application of fabricated system for drug delivery. c,d) Reproduced with permission.^[^ ^161^ ^]^ Copyright 2018, The Royal Society of Chemistry. e) Schematic diagram of micelles from synthesis, self‐assembly, to their application in vivo. Reproduced with permission.^[^ ^162^ ^]^ Copyright 2017, Wiley‐VCH. f) Micelles with ultralow critical micelle concentration to stabilize hydrophobic cargoes even in extremely diluted conditions. Reproduced with permission.^[^ ^163^ ^]^ Copyright 2018, Springer Nature. g) Electrically controlled gating system depending on the reversible wettability switch between superhydrophobic and superhydrophilic states. Reproduced with permission.^[^ ^164^ ^]^ Copyright 2017, Wiley‐VCH. h) Cumulative dye release of a tension‐responsive drug delivery system from electro‐spraying. Reproduced with permission.^[^ ^165^ ^]^ Copyright 2016, Wiley‐VCH.

Superhydrophobic materials are attracting great interest in constructing drug carriers for their potential in long‐term release.^[^ ^156^, ^157^ ^]^ For example, Manna et al. demonstrated that superhydrophobic multilayer coatings could extend the release process of small molecules for their excellent water repelling capacity;^[^ ^158^ ^]^ while Fan et al. constructed a type of superhydrophobic gated nanocontainers by loading diclofenac sodium (DS) into halloysite nanotubes (HNTs), followed by decoration with a polyorganosilanes layer, as shown in **Figure** **6**a.^[^ ^159^ ^]^ When the superhydrophobic system was placed into phosphate buffer solution of pH 7.4, the molecular release could be drastically slowed down compared to HNTs‐DS without superhydrophobic coating, which could be attributed to the decreased contact area by air cushion of Cassie state. Rather et al. synthesized a superhydrophobic polymeric matrix to coload two bioactive small molecules (DOX and tetracycline) which showed an extended corelease for over 6 months (Figure 6b).^[^ ^160^ ^]^ The polymeric material displayed a fairly high water CA of 158°, whereas the liquids with low surface tension such as ethanol could easily spread on the as‐described interface. With the evaporation of the ethanol, the excellent water repelling capacity of the polymeric matrix could be restored. As a result of this feature, DOX and tetracycline were easily coloaded into the superhydrophobic material. It was demonstrated that the released drugs from the polymeric material could effectively inhibit the proliferation of MG‐63 and MDA‐MB‐231 cancer cell lines as well as bacteria including *Staphylococcus aureus* (*S*. *aureus*) and *Escherichia coli* (*E*. *coli*), thus verifying the bioactivity of the selected molecules. More importantly, the release rate could be controlled by adjusting the wettability performance of the polymeric matrix through post chemical modification.

In addition, superhydrophilic biomaterials also exhibit indispensable position in improving the performance of drug carriers. For example, Cao et al. found that zwitterionic poly(carboxybetaine) with superhydrophilic property could stabilize the liposomes and thus, address the instability of conventional polyethylene glycol‐decorated ones.^[^ ^166^ ^]^ The superhydrophilic modification not only prolonged the blood circulating of drugs, but also exhibited excellent tumor inhibition effect in murine adenocarcinoma models. Cui and his coworkers developed a strategy to improve the cellular uptake by encapsulating hydrophobic DOX into highly hydrophilic and biodegradable polymers with guanidinium groups via *π*–*π* interaction, as shown in Figure 6c,d.^[^ ^161^ ^]^ When applied for in vitro cell experiments and in vivo animal tests, the self‐assembled conjugation system showed improved cellular uptake, real‐time drug release, and enhanced cancer therapy efficiency. To prolong the circulation period of liposome‐based drug delivery system, Nag et al. introduced a kind of superhydrophilic polymer to decorate liposome surfaces and evaluated their persistence.^[^ ^167^ ^]^ To be specific, the superhydrophilic polymer was conjugated with a lipid anchor N^1^‐(2‐aminoethyl)‐N^4^‐hexadecyl‐2‐tetradecylsuccinamide in this study to achieve enhanced circulation persistence in blood.

It is worth mentioning that the utilization of wettability contrast opens a new chapter in constructing drug carriers with stable characteristic, especially for the fabrication of micelles. Chen et al. proposed a linear diblock amphipathic polymer chain achieved from the polymerization of superhydrophilic poly(2‐methylacryloyloxyethyl phosphorylcholine) and a typical chemotherapy drug poly(10‐hydroxy‐camptothecin methacrylate), as shown in Figure 6e.^[^ ^162^ ^]^ The synthesized polymer chain could then be assembled into micelles which not only improved the hydrophobic drug loading efficiency, but also presented superhydrophilic outer surface to prolong the retention of hydrophobic drugs in blood. In addition, the polyprodrug amphiphile could be rapidly hydrolyzed in response to the esterase that extensively distributed in tumor sites and other specific tissues, thus enhancing the targeting capacity. Meanwhile, Lu et al. took advantage of the sharp contrast system, that is, molecules composed of superhydrophilic polymer domain and superhydrophobic domain, to address the instability problem of micelles (Figure 6f).^[^ ^163^ ^]^ Compared to conventional ones, the achieved sharp‐contrast micelles displayed an ultralow critical micelle concentration (CMC), indicating the capacity of maintaining their stability even at extreme dilution conditions. To verify the practical value of the ultralow‐CMC micelles, docetaxel formulation was encapsulated in the micelles and then injected into melanoma tumor‐model mice. The formulation showed unbelievable anticancer capacity that conventional drugs could not match, benefitting from the excellent stability of sharp‐contrast micelles.

Because of the tunable wetting behaviors in response to different stimulations, the wettability materials are playing an increasingly important role in constructing drug delivery system with controllable release property. Zhang et al. proposed an electrically controlled gating system for pulsatile drug delivery based on the wettability transitions between superhydrophobicity and superhydrophilicity states, as shown in Figure 6g.^[^ ^164^ ^]^ The gating system was constructed by the integration of perfluorooctanesulfonate ion‐doped polypyrrole layer with micro/nanoporous structure and an anodic aluminum oxide nanoporous film. Because of the reversible variation of redox potentials, the hydrophobic perfluorooctanesulfonate ions could be correspondingly released and back to the polypyrrole film, leading to the conversion between superhydrophobic and superhydrophilic property. To investigate the efficiency of the electrically actuated gating system in drug delivery, Penicillin G sodium and Rhodamine B aqueous solutions were, respectively, poured into a vessel equipped with a gating system at the bottom. When the polypyrrole film was in the reduction state (superhydrophilic state), the drugs could be continuously released; while the drug concentration virtually kept constant when the polypyrrole film was in the oxidation state (superhydrophobic state). The pulsatile drug release capacity of the switchable wettability system showed its great potential for on‐demand drug therapy.

Except for electrical stimuli, the release of wettability‐based drug carriers could also be controlled by other external forces. Wang et al. described a release system with pH‐responsive property based on the convertible wettability of mesoporous silica nanopores, which was originated from the attachment of phenylamine (Ph) groups that could convert their hydrophobic or hydrophilic states in the case of deprotonation and protonation.^[^ ^168^ ^]^ When the pH value of solution decreased, the Ph‐decorated nanopores would switch from hydrophobic status to hydrophilic behavior, thus releasing the encapsulated cargoes. In addition, Wang et al. reported a tension‐responsive drug delivery system that was composed of multilayered superhydrophobic microparticle coatings with drug loading by using electrospinning technique (Figure 6h).^[^ ^165^ ^]^ The drug release could be controlled by the crack propagation on the polymeric coating via applying strain of different magnitudes. In a later work by the same team, they established a tension‐activated superhydrophobic system to demonstrate the stimuli‐responsive release of small molecules and proteins.^[^ ^169^ ^]^ In general, wettability materials are ideal candidates for constructing drug carriers with high drug loading, prolonged circulation period, enhanced cellular uptake and so on, which may facilitate the clinical process of drug delivery systems.

### 4.3 Contrast Agents

**Figure 7:**
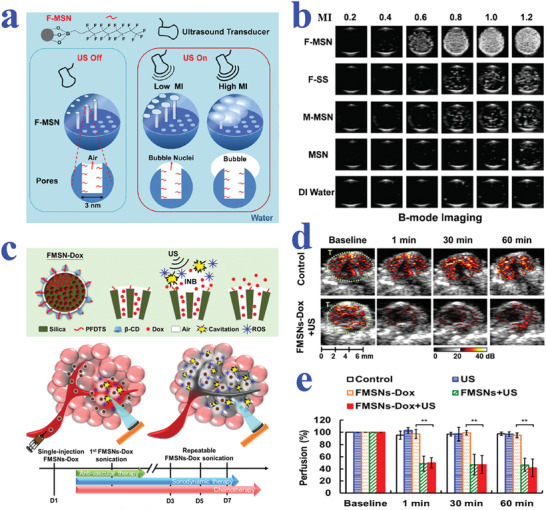
a) Mechanisms of bubble generation from superhydrophobic MSNs. Small bubble nuclei are formed at a lower mechanical index (MI) while big bubbles are generated at a high MI. b) Representative ultrasound images of various nanoparticles under different intensity of MIs. a,b) Reproduced with permission.^[^ ^170^ ^]^ Copyright 2016, Elsevier. c) Schematic diagram illustrating the generation of bubble cavitation, reactive oxygen species (ROS), and the release of DOX from superhydrophobic MSNs under ultrasound stimulation and their application in antivascular effect to increase intratumoral accumulation when injected in vivo. d) Tumor perfusion images for evaluating antivascular therapy of sonication. e) Statistical graph of the quantification for tumor perfusion degree. c‐e) Reproduced with permission.^[^ ^171^ ^]^ Copyright 2019, Elsevier.

Microbubbles have been widely utilized as contrast agents in ultrasound imaging, whose further application is limited by their instability, while superhydrophobic materials showed capacity in maintaining the existence of nanobubbles which could nucleate bubble formation by exposing to ultrasound and showing potential in constructing stable and high‐contrast agents.^[^ ^170^, ^171^ ^]^ For example, Jin et al. employed the interfacial nanobubbles adsorbed on superhydrophobic mesoporous silica nanoparticles (MSNs) to generate microbubbles in situ by applying an ultrasound transducer, as shown in **Figure** **7**a.^[^ ^170^ ^]^ The superhydrophobic MSNs were capable of maintaining the stability of the bubble‐precursors until their conversion into microbubbles under certain mechanical indexes. To verify the imaging capacity of the superhydrophobic MSNs, the B‐mode contrast intensity of nanoparticles with different morphologies and wettability behaviors were compared (Figure 7b). The results showed that MSNs with superhydrophobicity and porous property could enhance the contrast intensity. Ho et al. also utilized the superhydrophobic MSNs with DOX loading and *β*‐cyclodextrin coating for the chemo‐sonodynamic therapy (Figure 7c–e).^[^ ^171^ ^]^ The superhydrophobic decoration could prevent the drug from leakage and adsorb amounts of interfacial nanobubbles (INBs); while the *β*‐cyclodextrin layer could improve the dispersity of multifunctional MSNs in aqueous solutions. The cavitation of INBs not only enhanced ultrasound contrast, but also damaged tumor tissues and produced reactive oxygen species to exhibit antivascular effect which eventually facilitated drug penetration. In addition, the multifunctional MSNs also demonstrated sustained‐release capacity, thus improving the curative effect and reducing side effects.

### 4.4 Biocrystallization

The crystallization of biomolecules such as proteins and peptides is a fundamental and universal process to analyze their microstructures. In general, crystallization contains two basic procedures: nucleation and growth, during which process the formation of bio‐interfaces is a critical step. As a heterogeneous nucleation platform, superhydrophobic surfaces have demonstrated values in facilitating the biocrystallization process and endowing the products with high quality.^[^ ^172^, ^173^ ^]^ Especially the biomolecule‐assembled superhydrophobic surfaces have intrinsic advantages of biocompatibility, nontoxicity, and biodegradability, which make them suitable for biomedical applications. Based on the self‐assembly of lysozyme, Gao et al. fabricated a superhydrophobic surface and applied it for protein crystallization.^[^ ^172^ ^]^ It is worth mentioning that the strategy is appropriate for depositing phase‐transited protein on virtually arbitrary surfaces. Compared with non‐modified and normal hydrophobic surfaces, the lysozyme‐coated superhydrophobic one showed much superior crystallization efficiency of model protein. Wu et al. also took advantage of a superhydrophobic proteinaceous platform to facilitate preferential crystallization of proteins and peptides.^[^ ^173^ ^]^ These typical examples reveal that surfaces with special wettability would open a new chapter in fundamental research of biomolecules.

### 4.5 Wound Healing

Wound healing has become a global healthcare concern because of its universality and therapeutic impediments. During wound healing, many factors like bacterial infection may impede the recovery process and even cause severe complications. To solve these, researchers have dedicated to developing biomaterials with specific wettability, together with adding antibacterial ingredients for further treatment. Up to date, medical dressings based on biomaterials with specific wettability have demonstrated values in accelerating the wound healing process through removing excessive biofluid around wounds,^[^ ^96^, ^174^ ^]^ inhibiting adhesion of harmful biomolecules,^[^ ^174^, ^175^, ^176^, ^177^, ^178^, ^179^, ^180^, ^181^, ^182^, ^183^, ^184^, ^185^, ^186^, ^187^, ^188^, ^189^, ^190^, ^191^, ^192^, ^193^, ^194^, ^195^, ^196^, ^197^, ^198^, ^199^, ^200^, ^201^, ^202^, ^203^, ^204^, ^205^, ^206^, ^207^, ^208^, ^209^, ^210^, ^211^, ^212^, ^213^, ^214^, ^215^, ^216^, ^217^, ^218^, ^219^, ^220^, ^221^, ^222^, ^223^, ^224^, ^225^, ^226^, ^227^, ^228^, ^229^, ^230^, ^231^, ^232^, ^233^, ^234^ ^]^ and releasing therapeutic drugs or molecules.^[^ ^174^, ^175^, ^176^, ^177^, ^178^, ^179^, ^180^, ^235^, ^236^, ^237^ ^]^

Among these medical dressings, superhydrophobic surfaces have been extensively applied for wound treating because of their remarkable antibioadhesion characteristic. By employing electrospinning and electrospraying technique, Li et al. prepared a superhydrophobic membrane with antimicrobial peptide and curcumin encapsulation for burn treatment.^[^ ^175^ ^]^ With efficient control over bacteria invasion and inflammation, the multifunctional dressing was beneficial for wound healing. Yao et al. manufactured an omniphobic hydrogel membrane through microfluidics, which could resist bacterial infection under the combined action of its hydrophobicity and zinc ions.^[^ ^176^ ^]^ These results reveal the importance of high hydrophobicity in practical applications. In fact, superhydrophilic materials are also suitable to construct medical dressings. Biofluid such as sweat and wound exudate may overhydrate wounds under inappropriate treatment, and thus impede wound recovery process. To create an ideal environment for wound healing, Mayandi et al. described a superhydrophilic dressing by utilizing gelatin fibers with polydopamine (PDA) and *ε*‐polylysine (an antimicrobial ingredient).^[^ ^177^ ^]^ The derived dressing displayed outstanding effect on treating wounds because the superhydrophilic texture could remove wound exudates, while *ε*‐polylysine was capable of eliminating bacterial bioburden. However, attributed to their intrinsically hydrophilic performance, the superhydrophilic dressings may leave certain biofluid at the wound site.

**Figure 8:**
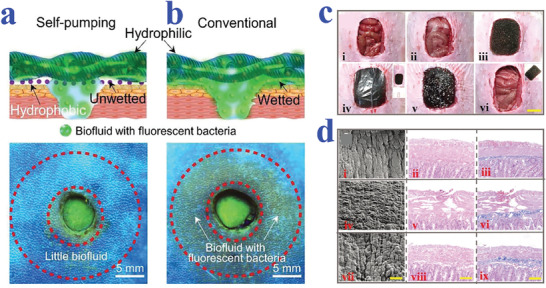
a,b) The wound condition after the treatment of self‐pumping dressing and conventional dressing to remove simulated biofluid, respectively.^[^ ^96^ ^]^ Copyright 2018, Wiley‐VCH. c) Wound repair process by employing slippery textile‐assisted therapy. d) Scanning electron microscopy and staining images of open‐abdomen models. Reproduced under the terms of the Creative Commons CC‐BY license.^[^ ^174^ ^]^ Copyright 2020, The Authors. Published by Wiley‐VCH.

The creation of materials with asymmetric wettability provides an effective solution for biofluid management.^[^ ^96^, ^178^, ^179^ ^]^ Shi et al. reported a self‐pumping dressing that is composed of hydrophobic nanofiber array and hydrophilic microfiber network (**Figure** **8**a,b).^[^ ^96^ ^]^ When the dressing was applied to the wounds, the excessive biofluid could be pumped from hydrophobic component to the hydrophilic side, thus preventing the wound from wetting and promoting its recovery. Recently, Yu et al. also fabricated a wound dressing with asymmetric wettability, favorable mechanical property, and high biocompatibility.^[^ ^178^ ^]^ The composite dressing was composed of a highly hydrophobic outer layer for preventing bacteria and a hydrophilic inner layer to promote tissue repair. Based on this design, the proposed dressing could enhance the healing of diabetic wounds.

Liquid‐infused biomaterials are also ideal candidates for wound dressing, owing to their superior antibiofouling performance. Zhang et al. described a slippery textile from microfluidic printing for medical drainage of wounds.^[^ ^174^ ^]^ Compared with common textiles, the paraffin‐infused slippery textile showed extraordinary liquid‐repellence. Based on this feature, the slippery textile‐assisted vacuum sealing treatment effectively facilitate the effusion drainage and wound repair (Figure 8c,d). When the slippery surfaces are loaded with antibacterial components, the resultant medical dressing could cater more complicated wound situation. For instance, Shi et al. developed a composite wound dressing by integrating an oil‐infused 3D printed polydimethylsiloxane (PDMS) layer with silver nanoparticles, which possessed antifouling, antiblood adhesion, and antibacterial properties for promoting infected wound healing.^[^ ^180^ ^]^ These examples prove the practical value of specifically wettable materials in constructing medical dressings for clinical treatment, and these materials would find wider applications in future with the innovation of technology.

## 5 Biosensing

Biosensing is of great significance to biomedical fields such as clinical diagnosis, biological analysis, and drug evaluation. Given that, a great variety of biosensors have been proposed to convert target‐molecule concentration into electrical signals, optical signals, or other visualized signals for quantification. However, it is difficult for traditional biosensors to further decrease the volume of sensing system and improve the detection sensitivity of analytes in diluted solutions. Therefore, novel biosensors with miniature dimension and high detection sensitivity are still anticipated. Benefitting from the advantages of droplet manipulation, wettability materials are rising stars in biosensing fields.^[^ ^182^, ^183^ ^]^ In particular, the combination of superhydrophobic substrate and superhydrophilic arrays provides a versatile platform for high‐throughput and low consumption detection. In addition, the extreme wettability contrast could enrich analysts from diluted solutions, which is conductive to increasing the detection sensitivity. In the following sections, we will introduce the research process of wettability materials with specific wetting behaviors in constructing biosensing devices for different applications, such as biomolecule detection including microRNAs (miRNAs), DNA, glucose, proteins, and antigens. Besides, biosensors based on wettability materials are also capable of serving as wearable electronics to monitor the exercise and health situation of human.

### 5.1 Detection of Biomolecules

#### 5.1.1 miRNAs Detection

miRNAs are playing an important role in various biological processes such as cell proliferation, metabolism, and even cancer development. Further researches have showed that the occurrence of cancer is usually companied with the higher or lower expression of miRNAs. Hence, miRNAs have become one of the most common biomarkers for clinical diagnosis. Tremendous efforts have been devoted to developing effective platforms for miRNAs detection such as microplates and suspended microspheres. Wettability materials are attaching great interest for the superiority in precise manipulation of motion, enriching of droplets based on specific wettability design, and high‐throughput property. In general, biomaterials of specific wettability are combined with advanced detection strategies such as surface‐enhanced Raman scattering (SERS) and fluorescence enhancement effect for miRNA quantification in higher selectivity and sensitivity as well as low consume manner.^[^ ^185^, ^186^, ^187^, ^188^ ^]^

**Figure 9:**
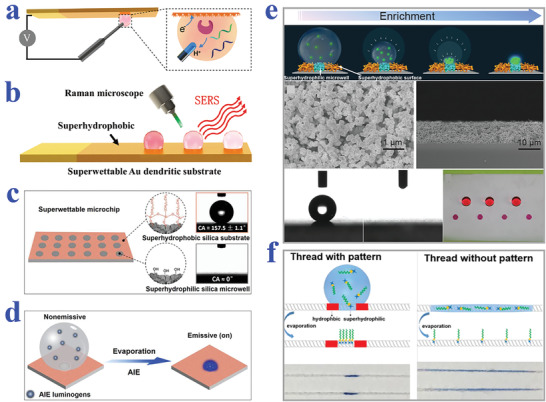
a) Schematic diagram showing electrochemical detection of the superwettable biosensor. Reproduced with permission.^[^ ^185^ ^]^ Copyright 2018, American Chemical Society. b) Schematic illustration of superwettable nanodendritic gold substrate for detecting multiple analytes based on SERS technology. Reproduced with permission.^[^ ^186^ ^]^ Copyright 2018, The Royal Society of Chemistry. c) The superhydrophobic substrate with superhydrophilic microwells. d) Transition of nonemissive AIE luminogens into emissive ones after evaporation on superwettable chip. c,d) Reproduced with permission.^[^ ^187^ ^]^ Copyright 2018, Elsevier. e) Condensing‐enrichment process of analytes on a superhydrophobic substrate with superhydrophilic patterns, owing to the wettability contrast. SEM images showing the top view and side view of nanodendritic silica coating. After OTS decoration, the superhydrophobic substrate was treated with UV irradiation to obtain superhydrophilic microwells. Reproduced with permission.^[^ ^189^ ^]^ Copyright 2015, Wiley‐VCH. f) Schematic illustration of the enrichment of probe molecules on the cotton thread with/without wettability patterns and corresponding images of a water droplet (containing blue dye) applied to the threads. Reproduced with permission.^[^ ^190^ ^]^ Copyright 2016, Elsevier.

Among a variety of them, electrical or electrochemical biosensors are recognized as popular choices for biological sensing because of their easy operation and short detection time. Xu et al. designed a superwettable electrochemical biosensor for versatile sensing of prostate cancer biomarkers based on electrochemical deposition and template‐assisted plasma etching techniques, as shown in **Figure** **9**a.^[^ ^185^ ^]^ The superhydrophobic/superhydrophilic micropatterns on the nanodendritic electrochemical biosensor could confine the sample droplets in superhydrophilic microwells, thus reducing the consume of sample solution. A series of cyclic voltammetry curves demonstrated that the droplet size and tilting angles had little impact on the results, which revealed the stability of the biosensor, while the contact area between droplets and the substrate showed positive relationship with the probe‐binding efficiency and signal magnitude. For the detection of miRNAs, the superwettable platform was composed of an electrode‐bound and redox‐reporter‐modified DNA probe. Once the targeting miRNAs were combined with DNA probe, the reporter would be kept away from the electrode surface because of the conformational change, thus leading to the variation in current signal. Based on the mechanism, the superwettable biosensor successfully realized sensitive and selective detection of cancer biomarkers involving miRNA‐375 and miRNA‐141, showing great potential in facilitating the development of portable sensing devices.

Compared with electrical biosensors, SERS‐based ones showed advantages of high sensitivity even when detecting highly diluted analytes. As a consequence, SERS technique has been attempted to integrate with wettability materials for improving the detection efficiency of miRNAs. Song et al. combined the SERS technology with superhydrophobic/superhydrophilic patterned nanodendritic gold substrate for the simultaneous detection of multiconcentration miRNAs, as shown in Figure 9b.^[^ ^186^ ^]^ The superhydrophilic arrays allowed the parallel and repeatable detection of multiple concentrations, while the nanodendritic gold could serve as rough SERS substrate for analytes detection in a highly sensitive manner. The droplets containing analytes could be captured and enriched by the superhydrophilic arrays to increase the detection limit. When applied for miRNA detection, DNA probe decorated by Raman molecule (Rhodamine X) was immobilized on the superhydrophilic region and hybridized with placeholder DNA. After exposed to target miRNA, the probe DNA would be replaced by miRNA, thus leading to the approach of Rhodamine X to the gold surface. In this case, low SERS intensity was switched into strong SERS signal for miRNA‐141 sensing, whose detection limit was 10^−12^ m. Although this instance displayed high sensitivity of SERS‐technique assisted wettability platform in biosensing, the detection precision still could not reach the clinical requirements.

Based on other detection strategies, many attempts have been made to construct wettability biosensors. Chen et al. described an aggregation‐induced emission (AIE)‐based superwettable platform with fluorescence enhancement for detection of miRNAs, as shown in Figure 9c,d.^[^ ^187^ ^]^ Because of the wettability contrast between superhydrophilic microwell and superhydrophobic substrate, droplets containing AIE luminogens could be confined and subsequently condensed in the superhydrophilic arrays after evaporation, thus inducing the fluorescence of AIEgens. Based on this feature, the detection of miRNA‐141 in real samples with high reproducibility, sensitivity, and specificity was realized on the AIE‐based superwettable platform. Besides, Cai et al. presented a condensing‐enriched superhydrophobic platform for multiplexed miRNA detection with the integration of magnetic photonic crystal barcodes.^[^ ^188^ ^]^ The emergence of wettability‐based biosensors has achieved some success in decreasing sample consumption, facilitating sample enrichment, and improving throughput, which provides a new direction for accurate biomolecule detection. However, these wettability‐based detection strategies are still in the initial stage, far from being mature for practical applications.

#### 5.1.2 DNA Detection

In addition to miRNA detection, the construction of DNA biosensors also has significance in various fields ranging from clinical diagnosis, drug development, to gene therapy. Over the past decades, the research emphasis was focused on improving the sensitivity of DNA biosensors by integrating signal transduction and signal amplification techniques. Among them, the common signal transduction techniques involve colorimetric, electrochemical, light scattering, and fluorescent approaches, while amplification strategies mainly include nanomaterial‐based signal amplification, enzyme catalysis, and molecular biology‐based amplification. To further enhance the detection sensitivity, wettability materials, especially the wettability‐patterned substrates are applied in DNA sensors for their capacity of enriching DNA samples from dilutions. With the assistance of condensing‐enrichment strategy, the DNA detection limit could be further reduced.

For instance, Xu et al. reported a biosensor based on superhydrophobic substrate with superhydrophilic microwells for ultratrace DNA sensing, as shown in Figure 9e.^[^ ^189^ ^]^ On the account of the wettability difference of the platform, the droplets containing trace analytes could be enriched on the superhydrophilic microwells to amplify the fluorescence signals, thus realizing the detection of DNA in a highly sensitive manner. However, the described superhydrophilic/superhydrophobic biosensor could not meet the requirement of sample flow during detection process. To overcome the shortage, the group developed a wettability‐patterned DNA biosensor on superhydrophilic cotton threads in a following work (Figure 9f).^[^ ^190^ ^]^ To enhance the enrichment of target molecules, the cotton thread was selectively blocked by liquid wax whose wettability showed temperature‐dependent property. When the temperature was below 19 °C, the wax modified regions presented hydrophobic behavior, in which case the sample droplet could be confined at superhydrophilic regions for the following detection process after evaporation. During the detection at room temperature, the wax would melt and keep hydrophilic property to ensure the flow of running buffer. Based on the specific wettability design and sandwich hybridization reaction, the DNA biosensor realized a detection limit of 0.75 nm. The cotton thread‐based biosensor displayed advantages of less sample demand, more rapid detection speed, and lower cost. In general, the employment of wettability materials provides a new development direction for point‐of‐care (POC) diagnostics of nucleic acid.

#### 5.1.3 Glucose Detection

**Figure 10:**
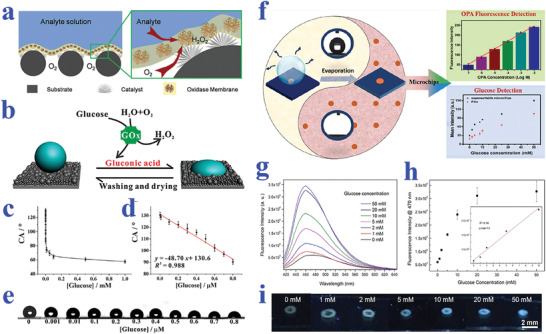
a) Schematic diagram of the superhydrophobic three‐phase biosensor: the electrode that modified with H_2_O_2_ catalyst is coated with an oxidase/chitosan layer. Reproduced with permission.^[^ ^191^ ^]^ Copyright 2015, Wiley‐VCH. b) Schematic illustration showing the reversible wettability transition of the substrate that caused by pH variation from GOx‐catalyzed reaction. c) Relationship between glucose concentration (from 1 nm to 1 mm) and CA values. d) Linear relationship between CA and glucose concentrations (from 1 nm to 800 nm). e) Wetting performances of droplets on the substrate under different glucose concentrations. b‐e) Reproduced with permission.^[^ ^193^ ^]^ Copyright 2018, Springer Nature. f) Illustration of superwettability‐patterned microchips for the detection of o‐phthalaldehyde and glucose. Reproduced with permission.^[^ ^194^ ^]^ Copyright 2019, American Chemical Society. g) Fluorescence spectra of evaporated droplets containing glucose of different concentrations on microchips. h) Relationship between fluorescence intensity at 470 nm and concentration of glucose. i) Fluorescent images of evaporated droplets containing glucose in fluorescent polydopamine (PDA) spots. g‐i) Reproduced with permission.^[^ ^195^ ^]^ Copyright 2018, American Chemical Society.

The accurate and rapid detection of glucose level is significant for the prevention and treatment of diabetes and other medical problems. The appearance of glucose sensors could be traced back to 1960s. Over the past decades, glucose biosensors have experienced great progress with various wettability‐based glucose biosensors having been developed. Among them, enzyme‐mediated glucose electrical/electrochemical biosensors have received extensive attention. For example, Lei et al. reported a superhydrophobic oxygen‐rich enzyme biosensor with solid–liquid–air three‐phase interface for glucose detection (**Figure** **10**a).^[^ ^191^ ^]^ The electrochemical biosensor was constructed by immobilizing glucose oxidase on Pt catalyst‐modified superhydrophobic substrate. When the resultant biosensor was applied for biosensing, the glucose concentration could be converted into current signal caused by electrooxidation of hydrogen peroxide (H_2_O_2_) which was generated from glucose oxidase catalyzed reaction. Because of the trapped air pockets on the electrodes, sufficient supply of oxygen was ensured during the detection process. According to the cyclic voltammograms, the concentration of glucose was proportional with the current whose linear detection range achieved 156 mm, far exceeding that of normal electrodes (about 5 mm). Hence, the introduction of superhydrophobic element imparted the biosensors with wider linear detection range, better sensitivity, and accuracy than conventional ones. Similarly, Guan et al. described an enhanced enzymatic reaction system with triphase gas–solid–liquid interface through using superhydrophobic mesoporous ZnO nanowire arrays.^[^ ^192^ ^]^ Benefitting from high diffusion coefficient of oxygen, the reaction kinetics could keep sustaining and constant to enhance the detection performance.

Although enzyme‐based glucose biosensors have achieved great progress, majority of them depend on complex equipment for analyzing, which impedes their popularization in population. To solve this problem, Gao et al. demonstrated a wettability‐based POC testing platform with pH‐responsive characteristic for non‐invasive detection of glucose, as shown in Figure 10b–e.^[^ ^193^ ^]^ In response to the increase of pH values, the POC platform could switch from superhydrophilic to superhydrophobic state. Additionally, the glucose concentration had a positive correlation with gluconic acid, owing to the glucose oxidase‐catalyzed reaction. Based on these principles, the CA would decrease with the increase of glucose concentration. In this case, the occurrence of diabetes could be diagnosed by simply monitoring the variation of CA without invasion and external instruments, and the results demonstrated higher accuracy than commercial glucometers. Besides, the platform showed advantages of excellent stability, durability, and versatility, which made it suitable for scale‐up production.

In addition to visual assays based on CA change, glucose biosensors based on fluorescent or colorimetric signals are also arousing great interest. For example, based on the oxidative color rendering capacity of glucose, Huang et al. developed a superwettability chip for glucose detection whose detection limit reached 2 mm, as shown in Figure 10f.^[^ ^194^ ^]^ The biosensor was designed with superhydrophilic/superhydrophobic micropatterns to anchor and enrich analyte solutions. During the detection process, the glucose concentration could be reflected by the H_2_O_2_ content generated from glucose oxidase reaction. By simply measuring the color intensity, the glucose content could be detected in a visual and sensitive way. Meanwhile, Beyazkilic et al. reported a superwetting platform as glucose biosensor depending on the fluorescent PDA (Figure 10g–i).^[^ ^195^ ^]^ The PDA emission could be enhanced by H_2_O_2_, the product of glucose oxidation reaction, providing a powerful tool for glucose detection. In addition, the enrichment capacity of superhydrophilic patterned superhydrophobic substrate imparted the biosensor with increased reaction rate and sensitivity. When the glucose concentration was within the range of 1–50 mm, bright PDA fluorescence could be observed and recognized by the naked eye. The strategy greatly simplifies the detection process, which may find its practical application in our daily life.

#### 5.1.4 Protein Detection

**Figure 11:**
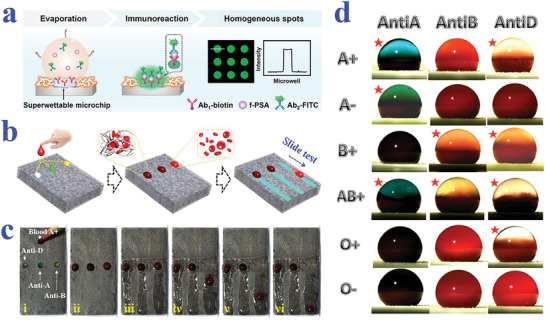
a) Detection of f‐PSA on superwettable microchips. Reproduced with permission.^[^ ^196^ ^]^ Copyright 2018, Elsevier. b) Schematic representation of visualized blood grouping on the intelligent slippery surface by simply observing the sliding states of droplets. c) Digital images showing the grouping progress of blood sample (A+), where only the droplets with no hemagglutination reaction would slide down. b,c) Reproduced with permission.^[^ ^47^ ^]^ Copyright 2018, American Association for the Advancement of Science. d) Blood agglutination (marked with red stars) of different blood samples (A+, A−, B+, AB+, O+ and O−) with different antibodies (Anti‐A, Anti‐B and Anti‐D) on a superhydrophobic surface for blood type. Reproduced with permission.^[^ ^198^ ^]^ Copyright 2013, Elsevier.

Protein is also one of the most important biomarkers in various fields including cancer and other disease diagnosis, blood grouping, and biological mechanism research. The emergence of biomaterials with specific wettability provides a kind of ideal platform for protein detection. For example, Chen et al. presented superwettable microchips for sensitive biosensing of prostate‐specific antigen (f‐PSA), as shown in **Figure** **11**a.^[^ ^196^ ^]^ The analyte droplets would be anchored on the superhydrophilic spots and the analytes could distribute homogeneously on the region, which could be attributed to the enhanced Marangoni effect of superwetting region and the suppressed outward flow by nanodendritic silica architecture. To verify the feasibility in biosensing, f‐PSA was chosen to demonstrate the proof of concept. During the process, microchips were immobilized with the biotinylated f‐PSA antibody after streptavidin decoration, followed by the addition of f‐PSA and FITC‐labeled f‐PSA antibody. With the immunoreaction and droplet evaporation, the concentration of f‐PSA could be presented by visualized fluorescence intensity, whose detection limit was obtained as low as 10 fg mL^−1^. It was worth mentioning that the superwettable microchips were also suitable for the detection of f‐PSA in clinical samples, owing to their extraordinary sensitivity and specificity compared to clinical assays. In addition, Gao et al. also described a superwettable chip for the detection of prostate specific antigen based on CA change and the optimized detection limit was up to 3.2 pg mL^−1^.^[^ ^197^ ^]^

As is well‐known, blood grouping depends on the detection of different antigens distributed on the surface of red blood cells. As a consequence, the accurate detection of antigen could be employed for blood testing. Specific wettability materials have demonstrated great value in constructing droplet microreactors for such biological assays. For example, Li et al. proposed a superhydrophobic surface for blood typing by observing hemagglutination reaction of blood samples (Figure 11d).^[^ ^198^ ^]^ In addition to superhydrophobic materials, slippery surfaces are also capable of creating microreactors for biological analysis. Wang et al. developed a slippery surface with programmable wettability by infusing paraffin into porous graphene film and demonstrated its value in blood grouping, as shown in Figure 11b,c.^[^ ^47^ ^]^ Thanks to the photothermal feature of graphene, together with the temperature‐responsive transition of paraffin between liquid and solid, the described surface could reversibly switch the slippery and rough status under NIR light control. By further integrating specific photomasks, the paraffin would selectively melt to form simple or complicated pathways for droplet sliding. Based on this feature, the intelligent surface was utilized for blood group diagnosis. To be specific, the blood samples were induced by first‐level pathways to react with three solutions containing different antibody, respectively. After sufficient hemagglutination reaction, the film was irradiated by NIR to create second‐level pathways, in which case the matched blood droplet would be pinned to the surface while the droplets with no hemagglutination would slide down. Through simply observing the pinning or sliding states, the detection of antigens of the red blood cells could be realized.

Ninno et al. reported a biosensor based on superhydrophobic materials for Fourier Transform‐Infrared (FTIR) detection of analytes in a sensitive and specific manner.^[^ ^199^ ^]^ With the assistance of superhydrophobic surfaces, the analyte solutions could be driven and enriched to the sensing focus of FTIR spectro‐microscope. In addition, the specially designed plasmonic nanoantenna arrays could significantly enhance electromagnetic field. Because of these two effects, the biosensor realized the detection of ferritin, a typical blood protein, in a label‐free, non‐destructive, and high sensitive (nanomolar level) manner. After purification treatment of samples, the biosensor could even measure the secondary structure of the protein, which may greatly facilitate the progress of clinical diagnosis such as early diagnosis of Alzheimer's disease. However, the FTIR‐based detection technology should depend on the usage of complex equipment, whereas colorimetric assays demonstrate unparalleled advantage in simple and visualized read out way. Xu et al. took advantage of superhydrophilic/superhydrophobic surfaces to serve as biosensing platform for the detection of protein.^[^ ^200^ ^]^ Due to the droplet capture capacity of superhydrophilic microwells, samples containing protein could be detected regardless of gravity. By simply observing color intensities and utilizing software for analysis, the concentration of protein could be quantified.

#### 5.1.5 Other Biomolecules Detection

Apart from the abovementioned nucleic acid, glucose, and protein, wettability‐based biosensors have also been developed for detecting other biomolecules such as urea,^[^ ^193^ ^]^ gluconolactone,^[^ ^201^ ^]^ malate,^[^ ^201^ ^]^ fumarate,^[^ ^201^ ^]^ histidine,^[^ ^201^ ^]^ phenylalanine,^[^ ^201^ ^]^ and even bacteria^[^ ^202^ ^]^ and cells.^[^ ^203^ ^]^ Based on pH responsive superwetting substrate together with urease‐catalyzed reaction, Gao et al. successfully achieved specific detection of urea molecule by naked eyes.^[^ ^193^ ^]^ Yu et al. developed a microfluidic chip by forming poly(ethylene glycol) hydrogel microwell arrays on a nanoporous aluminum oxide membrane for bacteria detection.^[^ ^202^ ^]^ Benefitting from the wettability difference, anti‐*E*. *coli* antibody could be effectively absorbed by the modified aluminum oxide substrate and be conducive to anchoring *E*. *coli*. Further impedance experiments demonstrated improved detection limit (10^2^ colony forming unit/mL) compared to conventional microelectrode array‐based method (10^4^ colony forming unit/mL). In addition, Park et al. realized the detection of cellular polarity depending on wettability‐responsive dyes.^[^ ^203^ ^]^ In general, specific wettability biomaterials provide a versatile platform for the detection of various biomolecules, and are conducive to enhancing the sensitivity, shortening reaction time plus reducing sample consume. These features make wettability materials ideal candidates for constructing next‐generation biosensors.

### 5.2 Wearable Biosensors

**Figure 12:**
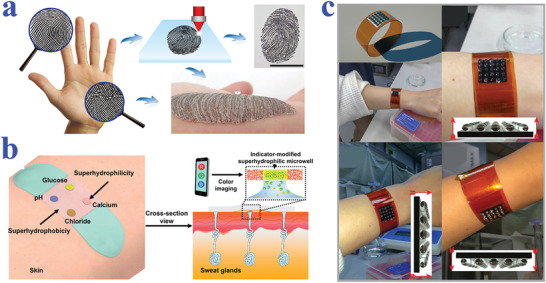
a) The fabrication of skin‐like wearable elastomer through direct laser writing technique. Reproduced with permission.^[^ ^204^ ^]^ Copyright 2018, Wiley‐VCH. b) Schematic diagram of superwettable bands with multiplex targets analysis capacity, companied with a cell‐phone screening module. Reproduced with permission.^[^ ^205^ ^]^ Copyright 2019, American Chemical Society. c) A wearable device worn on arm for water capture. Reproduced with permission.^[^ ^206^ ^]^ Copyright 2019, American Chemical Society.

With the development of science and technology, wearable electronics are gaining particular attention and interest in recent decades. In particular, wearable biosensors that can monitor human health situation by detecting the biological markers in sweat are anticipated. Many efforts have been devoted to developing flexible biosensors based on biomaterials with specific wettability. For example, Wang et al. developed one kind of skin‐like superhydrophobic elastomer materials that could switch wettability upon human motions (**Figure** **12**a).^[^ ^204^ ^]^ He et al. designed a kind of superwettability‐patterned bands for in situ perspiration biosensing, as shown in Figure 12b.^[^ ^205^ ^]^ The flexible bands were composed of three functional layers, including an adhesive layer attached to human skin, a superhydrophilic array layer integrated with sensing elements, and a superhydrophobic coating layer. Because of the wettability gradient, the sweat that was continuously secreted from human skin could be driven to the superhydrophilic regions and detected by the embedded colorimetric‐indicators. With the aid of cellphone, the superwettable bands were endowed with multiplexed biosensing ability covering pH, chloride, glucose, and calcium by utilizing colorimetric method. Owing to their simple design and low costs, the flexible and superwettable sensing system is suitable for mass production and pragmatic applications in personal health and clinical monitoring fields.

Li et al. reported an electrochemical biosensing system with wearable property for biomarker detection contained in sweat (Figure 12c).^[^ ^206^ ^]^ The wearable device was constructed by laser scribing of polyimide to generate superhydrophobic substrate with superhydrophilic graphene arrays. Thanks to the wettability gradient design and excellent conductivity of graphene, the derived biosensor could realize effective capture and electrochemical measurement of sweat droplets even in motion. When the device was directly applied to human skin, it demonstrated favorable attachment performance and sample collection capacity during exercise for the following detection. By adding glucose oxidase to the graphene electrode, the biosensor device could realize precise detection of glucose concentration according to the current change induced by H_2_O_2_ molecules that was generated from reaction. Although the graphene‐integrated biosensors show great potential in serving as wearable monitoring devices, they have defects in limited sensitivity and dependence on complex analysis equipment. In short, the introduction of special wettability materials facilitates the development of flexible and integrated biosensors, and future effort could be focused on improving the sensitivity, reducing the volume, and decreasing the cost.

## 6 Medical Apparatus and Instruments

The development of medicine field raises a higher demand to the functionality and stability of biomaterials for their indispensable roles in constructing medical apparatus and instruments. However, complex physiological environment of human body would break down the properties of materials through unwished adhesion such as thrombocyte attachment and bacterial infection, and thus complications that may threaten human health would be induced. In particular, biomaterials in long‐term contact with human body like medical implants have higher requirements for resisting the adhesion of physiological fluids, proteins, microorganisms, and cells. To meet these demands, endless efforts have been devoted to designing the surface topographies and chemistry of biomaterials to impart them with excellent antifouling capacity and certain mechanical stability. Among them, superwetting materials including superhydrophobic, superoleophobic, and superamphiphobic ones have been applied to medical devices and achieved some success.^[^ ^35^, ^207^ ^]^ Besides, the emergence of liquid‐infused slippery surfaces provides another ideal choice for medical devices because of their defect‐free and durable wettability performance.^[^ ^41^ ^]^ In the following sections, we will put our emphasis on introducing the application of these biomaterials with specific wettability in constructing medical apparatus and instruments involving surgical instruments, medical tubing, and implants.

### 6.1 Surgical Instruments

Surgical instruments are playing an important role in modern medicine field. Because of the direct contact with human body, surgical instruments should be equipped with characteristics of certain mechanical strength, antifouling property, and prevention of cross‐contamination. Various antimicrobial or antifouling strategies have been developed and applied in surgical instruments to resist protein attachment, cell adhesion, and biofilm formation, such as metal‐ion based coatings, antifouling polymer modifications, and the application of antibacterial agents. Among them, superwetting coatings are receiving extensive attention for their excellent antibiofouling performances by simply adjusting surface energy and topography.^[^ ^208^ ^]^ For example, Ren et al. developed a kind of CuO nanoparticle‐contained superhydrophobic coating with excellent bacteria anti‐adhesion capacity and bactericidal performance.^[^ ^209^ ^]^ Zeng et al. also fabricated a multifunctional superhydrophobic coating of mechanical durability, antibacterial, and blood‐repellent properties, which showed great potential in constructing medical instruments.^[^ ^210^ ^]^ In addition to bacteria and blood resistance, as well as manipulation of cell behavior that is mentioned in the above sections, superhydrophobic substrate also has impact on repelling protein adhesion.^[^ ^211^ ^]^ Wang et al. even demonstrated protein patterning on a superhydrophobic substrate, with the assistance of magnetic field.^[^ ^212^ ^]^ These instances do illustrate the potential of superhydrophobic materials to serve as functional coatings.

**Figure 13:**
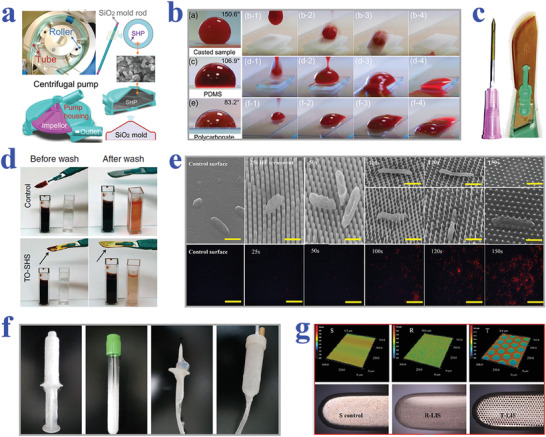
a) Fabrication of a superhydrophobic centrifugal pump housing, together with a roller blood pump tube from sand‐casting method. b) Blood resistance capacity of the sand‐casted superhydrophobic material, PDMS surface, and polycarbonate surface, respectively. a,b) Reproduced with permission.^[^ ^213^ ^]^ Copyright 2018, The Royal Society of Chemistry. c) A surgical‐grade steel scalpel blade and syringe needle coated with superhydrophobic tungstite oxide layer. d) Blood staining on the surface of untreated scalpels and scalpels with superhydrophobic coating before and after washing. c,d) Reproduced with permission.^[^ ^214^ ^]^ Copyright 2015, Springer Nature. e) SEM images of bacterial cell (*Escherichia coli*) morphology and fluorescent images of dead cells (stained red) on nanopillar‐structured quartz surfaces treated with HF solution for different times. Scale bars are 1 µm. Reproduced with permission.^[^ ^215^ ^]^ Copyright 2018, Elsevier. f) Digital images showing the versatility of solution blow spinning stratagem for coating different shaped objects: from left to right are syringe, evacuated blood tube, closure‐piercing device, and drip infusion device, successively. Reproduced with permission.^[^ ^218^ ^]^ Copyright 2016, American Chemical Society. g) 3D laser scanning images of intact monopolar scalpel, chemical etched rough scalpel, and textured scalpel surface, as well as their corresponding optical images. Reproduced with permission.^[^ ^219^ ^]^ Copyright 2018, American Chemical Society.

Based on the advances, various attempts have been made to verify the practical values of superhydrophobic coatings in medical instruments. Li et al. took advantage of sand‐casting strategy to prepare superhydrophobic surfaces with properties of blood‐repellence, durability, and flexibility (**Figure** **13**a,b).^[^ ^213^ ^]^ Attractively, the strategy was applicable to fabricate 3D superhydrophobic geometries, and the derived surface could effectively decrease water drag force, which made it ideal for improving the lifetime of medical devices such as blood pumps. Tesler et al. developed a superhydrophobic electrodeposited tungstite oxide film with antibiofouling performance and applied it to surgical instruments including scalpel blade and syringe needle, as shown in Figure 13c.^[^ ^214^ ^]^ The superhydrophobic property was gained from electrodepositing tungstite oxide layer on steel surfaces, followed by a fluorination treatment process. This technique could be conducted to steel surfaces of various grades, even medical‐grade ones. After modification, the decorated surgical instruments not only showed resistance to marine algal and *E*. *coli* adhesion, but also exhibited favorable blood repellence capacity (Figure 13d). These instances prove the practical value of superhydrophobic coatings in constructing medical devices.

Although superhydrophobic coatings have demonstrated values in antibiofouling, they are not applicable to surgical instruments that require favorable wetting property such as endoscopes. With this regard, superhydrophilic coating with bactericidal properties has received considerable interest and research. Early on, researchers have found that the nanopillar‐structured surface topography of the cicada wing could penetrate cell membranes and thus kill microorganisms. Inspired by this biological mechanism, Han et al. constructed a superhydrophilic surface with nanopillars to prevent the formation of biofilms on medical devices.^[^ ^215^ ^]^ The proposed superwettable surface demonstrated excellent killing ability on Gram‐negative bacterial species such as *Pseudomonas aeruginosa* (*P*. *aeruginosa*) and *E*. *coli*, as shown in Figure 13e. More importantly, the nanopillar‐structured superhydrophilic surface also displayed favorable antifogging and antireflective performances, which made it ideal for constructing optical devices in medical field. In general, superwetting materials are potential candidates to enhance the antibacterial and blood‐compatible properties of medical devices, thus reducing the risk of inflammation and infection in clinical treatment.

Despite some success, these strategies based on superwetting solid surfaces fail to maintain anti‐adhesion capacity in an effective and long‐term manner, which impedes their practical applications in medical field. In previous researches, the slippery liquid‐infused surfaces have shown robust biofouling resistance, which provides a new strategy for functionalizing surgical instruments. Compared with solid surfaces, the liquid‐infused surfaces demonstrate superiority in self‐repairing and stability. Therefore, SLIPS are promising materials to construct surgical instruments with robust antibiofouling capacity. For example, Epstein et al. found that SLIPS could prevent the attachment of various bacteria such as *P*. *aeruginosa*, *S*. *aureus*, and *E*. *coli* for a relatively long period, which was more superior than polytetrafluoroethylene and majority of superhydrophobic surfaces.^[^ ^216^ ^]^ Chen et al. proposed one typical medical biomaterial polytetrafluoroethylene with perfluorocarbon lubricant infusion and tested its performance in suppressing the formation of biofilms.^[^ ^217^ ^]^ In vivo experiments demonstrated that the derived SLIPS successfully resisted bacteria adhesion and decreased inflammatory response.

In addition to preventing bacteria attachment, SLIPS also have advantages over resisting protein and blood platelet adhesion. Yuan et al. fabricated a slippery microfiber coating which not only resisted the attachment of *P*. *aeruginosa*, but also showed repellence to blood components like platelets and erythrocytes. More importantly, the solution blow spinning strategy used in this study was suitable for modifying objects with different geometrical morphology (Figure 13f).^[^ ^218^ ^]^ Zhang et al. developed liquid‐infused electrosurgical instruments based on microcontact printing technique and demonstrated their excellent anti‐adhesion capacity, as shown in Figure 13g.^[^ ^219^ ^]^ With this feature, the electrosurgical instrument not only effectively decreased thermal damage and the resulted wound area, but also had potential values in bacteria and blood resistance. The introduction of SLIPS into surgical instruments greatly improves their performance, thus reducing the risks during surgical process and decreasing infection risk after treatment. It is also worth mentioning that SLIPS are suitable for creating specific medical instruments like camera‐guided ones, which could be attributed to the optical transparency of SLIPS.^[^ ^220^ ^]^ Generally speaking, the rapid developments of superwetting materials and SLIPS point out the direction for the construction of surgical instruments.

### 6.2 Medical Catheters

Medical catheters such as guiding catheters, hemodialysis catheters, venous catheters, and drainage tubes are essential and indispensable components of medical apparatus and instruments. Similar to surgical instruments, medical catheters usually require direct contact with organisms or physiological fluids. Among them, specific catheters like central venous catheters should stay in vivo for a relative long‐term period. Once the medical tubes are contaminated or blocked, the patients may suffer from severe infection risks which threaten their lives. Therefore, it is imperative to impart catheter materials with robust biocompatibility, anticoagulation, antibacterial, and anti‐adhesion capacities. Taking these into consideration, the development of interface science injects new vitality to the improvement of medical catheters. Over the past decades, researchers have attempted to modify the surface topography and chemical composition of medical catheters through physical or chemical methods, aiming at enhancing their performances. According to wettability performance, the modified catheters could be classified into two categories, that is, superwetting ones and slippery ones.

**Figure 14:**
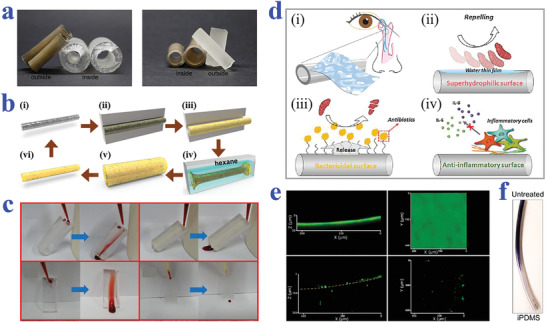
a) Superhydrophobic tubes with varying diameters from roll‐to‐roll manufacture. Reproduced with permission.^[^ ^223^ ^]^ Copyright 2016, Wiley‐VCH. b) Illustration of replication and detachment process for preparing superhydrophobic PDMS tubes. Reproduced with permission.^[^ ^224^ ^]^ Copyright 2019, Elsevier. c) Blood repellency capacity of different materials: smooth tube, superhydrophobic tube, smooth surface, and superhydrophobic surface, respectively. Reproduced with permission.^[^ ^225^ ^]^ Copyright 2020, Elsevier. d) Schematic diagram of superhydrophilic tubes with antibacterial and anti‐inflammatory functions for ophthalmic application. Reproduced with permission.^[^ ^226^ ^]^ Copyright 2018, Elsevier. e,f) Confocal microscopy images and optical image showing the formation of bacterial biofilms (*P*. *aeruginosa*) on untreated tubes and infused silicone tubing, respectively. Reproduced with permission.^[^ ^228^ ^]^ Copyright 2014, American Chemical Society.

Because of their superiority in resisting physiological fluids such as blood, urine, and saliva, superhydrophobic materials have received numerous attention and researches to investigate their values in constructing medical tubes. In addition, superhydrophobic tubes also have demonstrated effects on reducing blood hemolysis during extracorporeal circulation.^[^ ^221^, ^222^ ^]^ With these efforts, a variety of fabrication methods have been proposed to prepare superhydrophobic catheters. For example, Nokes et al. described a roll‐to‐roll processing method, which could be employed for high throughput generation of superhydrophobic plastics (**Figure** **14**a).^[^ ^223^ ^]^ The formed superhydrophobic surface could effectively prevent blood coagulation and be shaped into tubes of various diameters, which made it suitable for medical applications. Kim et al. reported a facile replication strategy to produce superhydrophobic tubes by taking PDMS as raw material, as shown in Figure 14b.^[^ ^224^ ^]^ Based on the swelling‐driven detachment principle, PDMS tubes could be easily separated from the micro/nanostructured aluminum mold. The as‐prepared inner surface of PDMS tubes exhibited superhydrophobicity, which could be attributed to the intrinsic hydrophobicity of PDMS and the hierarchical structure replicated from mold. Benefitting from the outstanding physical properties of PDMS, the superhydrophobic tubes were also in possession of high transparency and flexibility. More importantly, compared with normal tubes, the superhydrophobic polymer ones showed increased mass flow rate under pressure (5 kPa) and favorable blood repellence performance. Similarly, Wang et al. prepared PDMS superhydrophobic tubes by template method and demonstrated their values in repelling water and blood (Figure 14c).^[^ ^225^ ^]^

Although some progresses have been made, superhydrophobic catheters have defects in maintaining anti‐adhesion performance for a long term. As a replacement, superhydrophilic materials could effectively and stably resist the adhesion of bacteria and the formation of biofilm, which may be attributed to formation of a thin water film on their surface. More importantly, superhydrophilic catheters show advantages of favorable liquid compatibility, biocompatibility, and antifogging property. With this regard, superhydrophilic catheters are considered as ideal candidates for clinical applications. Park et al. developed a kind of superhydrophilic coating to decorate silicone tubes for ophthalmic applications, as shown in Figure 14d.^[^ ^226^ ^]^ To be specific, the superhydrophilic coating was fabricated by LBL assembly of polysaccharide film and a subsequent chemical cross‐linking. Because of the introduction of superhydrophilic layer, the derived silicone tubes exhibited antibacterial capacity and anti‐inflammatory performance. In addition, the superhydrophilic coating also demonstrated values in drug delivery, which greatly expands their prospects in medical treatment. Recently, Li et al. reported and demonstrated the value of a superhydrophilic coating from the formation of polydopamine and silver nanoparticles on catheters in antibacterial and antithrombotic aspects.^[^ ^227^ ^]^ The future directions may be focused on simplifying the production procedures and decreasing production costs of superhydrophilic materials, as well as further investigating their practical values in serving as medical catheters.

Apart from superwetting catheters, slippery materials are emerging and promising for constructing medical catheters. Since the concept of SLIPS was proposed by Aizenberg, abundant researches and developments correlating with this have emerged up during the past decade. It is demonstrated that SLIPS could achieve excellent antibiofouling performance and optical property based on appropriate design. More attractively, SLIPS are manufactured by infiltrating lubricating fluids into micro‐ or nano‐structured surfaces, which indicates their versatility for numerous substrates including medical tubes. Therefore, SLIPS have a wide horizon of developments in constructing medical catheters. For instance, MacCallum et al. described a liquid‐infused silicone to serve as biofouling‐free medical material, as shown in Figure 14e,f.^[^ ^228^ ^]^ Under the same conditions, a thick and robust *P*. *aeruginosa* biofilm formed on the untreated silicone tubing, while just few bacterial aggregates appeared on the surface of lubricant‐infused silicone tubing which could be easily removed after washing for seconds. The results revealed that the liquid‐infused strategy not only inhibited the initial formation of biofilms, but also helped in the removal of formed biofouling.

### 6.3 Implants

With the rapid developments of medical fields, multifarious implants such as artificial joint, cardiac valves, dental implants, and blood pumps have been developed and applied to human body for maintaining normal physiological functions. Because of the long‐time exposure to complex physiological environment, the medical implants composed of synthetic or natural materials should possess excellent biosecurity, stress tolerance, antibacterial property, as well as favorable repellence to the adhesion of biological molecules including proteins and blood platelets. Otherwise, the attachment of these biological components may induce severe inflammatory response that would be harmful to human health. To minimize the mentioned drawbacks, plentiful surface modification strategies have been proposed to decorate the medical implants for better compatibility. Among them, the introduction of superwettability or liquids has shown their effectiveness in improving the performance of medical implants.

As common implant materials, metallic implants involving stainless steel, titanium (Ti), magnesium, and cobalt (Co)‐base alloys ones have been widely applied in clinical applications. A tremendous amount of work is dedicated to investigating the influence of specific wettability decoration on the metallic implants. Moradi et al. explored the platelet adhesion behavior on stainless steel and Ti substrates with different wettability ranging from superhydrophobicity to superhydrophilicity.^[^ ^229^ ^]^ The results demonstrated that surface topography and chemical modification had great influence on the adhesion of platelets. When the substrates were in Cassie–Baxter status, they could effectively resist biological fluids. In addition, Liu et al. investigated the wettability influence of magnesium alloy on anticorrosion when they were employed as biomedical implants.^[^ ^230^ ^]^ To simulate the internal environment of organisms, Hank's simulated body fluid was chosen as model fluid for testing anticorrosion performance of magnesium alloy with different wettability. Among them, the superhydrophobic samples exhibited distinctive anticorrosion capacity, which might expand their clinical applications.

**Figure 15:**
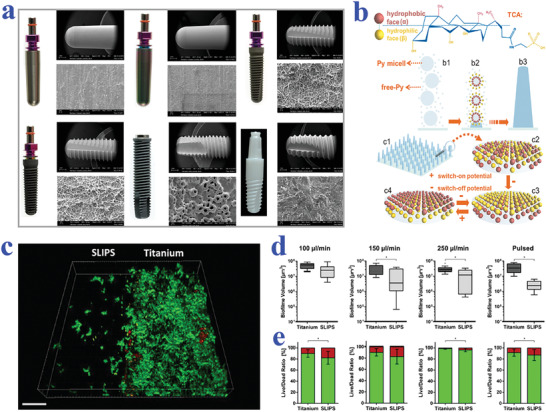
a) Digital images and corresponding SEM micrographs of different dental implants. Reproduced with permission.^[^ ^231^ ^]^ Copyright 2015, Elsevier. b) Chair formation of taurocholic acid and the possible mechanism illustrating its switchable wettability upon electrical potential. Reproduced with permission.^[^ ^238^ ^]^ Copyright 2014, Wiley‐VCH. c) 3D fluorescence images of bacteria adhesion on SLIPS and untreated titanium surfaces after pulsed washing. Scale bar is 200 µm. d) *S*. *oralis* biofilm volume and e) live/dead distribution graphs on SLIPS and untreated titanium surfaces under different washing velocities in a flow chamber system. c,d) Reproduced with permission.^[^ ^239^ ^]^ Copyright 2019, American Chemical Society.

Based on their superior performances, metallic materials with specific wettability have found wide applications in dental implants, bone implants, ophthalmic implants, etc. Thereinto, dental implants are becoming relatively mature, whose application could be traced back to ancient times. Nowadays, the rapid development of economy raises higher requirements for the omni‐directional performances of dental implants. Qahtani et al. explored UV‐induced wettability variation of screw‐type dental implants, as shown in **Figure** **15**a.^[^ ^231^ ^]^ Before exposure, all the implants presented hydrophobic property. Under the irradiation of UV‐A, merely the anatase‐decorated implants converted their original hydrophobicity into superhydrophilicity. When exposed to UV‐C, the grit‐blasted or acid‐etched implants would become superhydrophilic; while the anodized Ti and zirconia ones became hydrophilic, with the polyetheretherketone ones experiencing a slight hydrophilization. According to these results, the UV irradiation could enhance the wettability of dental implants, whose efficiency was related to the intrinsic material property and its surface. Based on the photo‐functionalization principle, Avila et al. described that UV‐treated Ti surface exhibited superhydrophilic status and effectively reduced the attachment of human oral bacteria, which shed a light on the development of dental implants.^[^ ^232^ ^]^

Bone implants, as the name implies, are commonly applied to replace and support missing or damaged bones and joints. Therefore, bone implants should be equipped with strong mechanical strength and anticorrosion properties. With this regard, tremendous efforts have been committed to functionalize metallic materials that have intrinsic strength, antifouling performance, and biocompatibility. Qin et al. demonstrated that wettability based on appropriate morphology design and chemical modification could effectively enhance the tribological performance of Co‐Cr‐Co alloy implants.^[^ ^233^ ^]^ Besides, Toita et al. reported a Ti implant with superhydrophilicity that was functionalized by ozone gas.^[^ ^234^ ^]^ Attractively, the resulting implant had impact on modulating bone marrow cells and even suppressing inflammation response. In addition, Lorenzetti et al. synthesized a nanocrystalline titania‐anatase coating on Ti bone implants through hydrothermal treatment, which showed extraordinary bacteria‐repelling capacity under the synergistic effect of photocatalysis and photo‐induced wettability.^[^ ^240^ ^]^

For better control over biological activities, responsive elements are integrated on the surface of implants.^[^ ^241^ ^]^ Liao et al. imparted Ti‐based bone implants with switchable wettability to control protein adsorption, as shown in Figure 15b.^[^ ^238^ ^]^ Through doping amphiphilic taurocholic acid into nanostructured polypyrrole array, the fabricated surface was capable of reversibly switching its wettability upon electrical potential variation, ranging between superhydrophobicity (152°) and hydrophilicity (55°). Based on the switchable wettability, the implant materials realized controllable protein adsorption and osteoblast adhesion, which set a solid foundation for their practical applications. Moreover, Zhu et al. reported an intelligent orthopedic implant to regulate bioactivity in situ under electrical control, which was caused by the nanostructured polypyrrole/glycosaminoglycans polymerized on the alloyed surface.^[^ ^242^ ^]^ Benefitting by this design, the wettability of the surface could be continuously and reversibly switched upon electrical stimulus, thus realizing the control over protein adhesion. These examples reveal the increasing importance of extreme wettability in metallic implants.

In addition to superwetting ones, lubricant‐infused materials have also displayed great potentials in improving the properties of metallic implants. As mentioned in the above sections, slippery surfaces are capable of effectively resisting the adhesion of microorganisms, blood platelets, and cells, which opens a new chapter for the construction of medical implants. For example, Doll et al. proposed a laser‐structured SLIPS based on Ti substrate, which showed great values in repelling the adhesion of *Streptococcus oralis*, human fibroblasts, and osteoblasts.^[^ ^243^ ^]^ In their following work, they integrated the Ti‐based SLIPS in an oral saliva‐simulated flow chamber system to verify its effect on inhibiting biofilm fouling. Compared with Ti control group, the liquid‐infused ones exhibited markedly reduced bacterial colonization, as shown in Figure 15c–e.^[^ ^239^ ^]^ Further cell force and gene sequencing experiments revealed that the underlying mechanism of antifouling performance was derived from the weakened bacteria adhesion to the substrate. In general, biomaterials with specific wettability are playing an increasingly important role in medical implants. However, the durable and robust performance of these implants still needs improvements to meet up with requirements of clinical applications.

### 6.4 Diagnostic Instruments

In modern medical treatment, diagnostic instruments such as endoscopes become increasingly significant.^[^ ^235^, ^236^, ^237^ ^]^ However, the lens would be polluted by body fluids during the operation, thus impeding the accuracy and precision, and even threatening the life of patients. In view of this, researches have devoted much effort to improving the antifouling capacity of diagnostic instruments. For instance, Tenjimbayashi et al. reported a facile as well as versatile strategy for the preparation of slippery coating, and applied it to endoscope lens as a proof‐of‐concept.^[^ ^235^ ^]^ The uncoated endoscope would lose its vision, which was attributed to the adhesion of water. By contrast, the slippery layer‐coated endoscope maintained clear vision field because of outstanding liquid‐repelling performance. Similarly, Sunny et al. introduced a transparent slippery coating on bronchoscope lens to enable an undisturbed field of view for expanding their practical prospects in medical treatment.^[^ ^236^ ^]^ In a word, the emerging of biomaterials with specific wettability opens a new chapter for the construction of medical devices and diagnostic instruments with more superior characteristics.

## 7 Conclusions and Perspectives

In this review, we have summarized the research progress on the biomaterials with specific wettability, ranging from the underlying mechanisms, diverse fabrication strategies to their application potentials. With the growing understanding on the mechanisms of wetting phenomenon and the advances in technology, numerous wettability materials with distinctive morphology design and functions are constantly emerging. It has been demonstrated that these wettability materials have broad prospects in biomedical engineering field such as tissue engineering, biosensing, and serving as medical devices.

Despite much exciting and impressive progress, there are still several critical issues to be considered and addressed for the improvements of wettability materials. The first issue refers to the construction of wettability substrates with more controllable and intelligent performances. One promising strategy is to impart the surface of substrates with switchable morphologies in response to external control, thus realizing the desired wettability variation. In addition, the substrate could be further coated or decorated with intelligent chemical molecules that have switchable wettability performance upon stimulations.

The second issue concerns the extension of these wettability materials’ biomedical applications. From the aspect of biomedical engineering, biomedical materials are designed to prevent, diagnose, treat, and rehabilitate human diseases. To achieve this, attention should be paid to maintaining the wettability performances, strengthening the mechanical property, improving the biocompatibility, simplifying the operation procedures, reducing the fabrication costs, and so on, through rationally integrating multidiscipline techniques such as physics, surface chemistry, biology, and medical field.

The third issue is in regard to the industrialization and marketization of these biomaterials with specific wettability. Majority of these materials still remain in the proof‐of‐concept stage, which has a long way toward practical applications. Given this, the controllability, intelligence, durability, flexibility, and biosafety of the wettability materials should be enhanced to transform them into commercial products. The collective efforts of researchers, entrepreneurs, and even medical professionals would play an important role in accelerating this process.

In a word, materials with specific wettability have accomplished great achievements in numerous fields like biomedical engineering. Future attempts should not only focus on the fundamental researches and technical innovation, but also promote the practical applications and explore commercial values of these materials. We expect this review would facilitate the communication and cooperation among multiple disciplines, thus giving birth to new sparkles for the development of wettability materials.

## Conflict of Interest

The authors declare no conflict of interest.
